# Assessing magnetic and inductive thermal properties of various surfactants functionalised Fe_3_O_4_ nanoparticles for hyperthermia

**DOI:** 10.1038/s41598-020-71703-6

**Published:** 2020-09-22

**Authors:** Arunima Rajan, Madhulika Sharma, Niroj Kumar Sahu

**Affiliations:** 1 Centre for Nanotechnology Research, Vellore Institute of Technology, Vellore, 632014 India; 2 School of Advanced Sciences, Vellore Institute of Technology, Vellore, 632014 India; 3 Department of Metallurgical Engineering and Material Science, IIT Bombay, Powai, Mumbai, 400076 India

**Keywords:** Nanoscale biophysics, Physics

## Abstract

This work reports the fabrication of magnetite (Fe_3_O_4_) nanoparticles (NPs) coated with various biocompatible surfactants such as glutamic acid (GA), citric acid (CA), polyethylene glycol (PEG), polyvinylpyrrolidine (PVP), ethylene diamine (EDA) and cetyl-trimethyl ammonium bromide (CTAB) via co-precipitation method and their comparative inductive heating ability for hyperthermia (HT) applications. X-ray and electron diffraction analyses validated the formation of well crystallined inverse spinel structured Fe_3_O_4_ NPs (crystallite size of ~ 8–10 nm). Magnetic studies confirmed the superparamagnetic (SPM) behaviour for all the NPs with substantial magnetisation (63–68 emu/g) and enhanced magnetic susceptibility is attributed to the greater number of occupations of Fe^2+^ ions in the lattice as revealed by X-ray photoelectron spectroscopy (XPS). Moreover, distinctive heating response (specific absorption rate, SAR from 130 to 44 W/g) of NPs with similar size and magnetisation is observed. The present study was successful in establishing a direct correlation between relaxation time (~ 9.42–15.92 ns) and heating efficiency of each surface functionalised NPs. Moreover, heat dissipated in different surface grafted NPs is found to be dependent on magnetic susceptibility, magnetic anisotropy and magnetic relaxation time. These results open very promising avenues to design surface functionalised magnetite NPs for effective HT applications.

## Introduction

Magnetic hyperthermia (MHT) is one of the most propitious minimally invasive therapies for carcinogenic cell ablation^1,2^. Aqueous stable biocompatible magnetic nanoparticles (MNPs), guided by an external magnetic field can potentially release heat selectively at the carcinogenic tumor sites and are often employed as efficient heat mediators for MHT^3,4^. One of the key parameters to be monitored for a successful clinical outcome is to achieve the desired therapeutic temperature (42–46 °C) with minimal dosage of MNPs^5^. In this regard, superparamagnetic iron oxide nanoparticles (SPIONs) are receiving increasing attention. SPIONs possess the potentiality to be directed by an AC magnetic field in order to specifically target the tumor regions and to dissipate heat locally. Superparamagnetism (SPM) results in negligible agglomeration between particles (feeble magnetic interactions) after the removal of field causing trivial side effects in the body^6,7^. The SPM behaviour of NPs is very crucial for HT applications as these MNPs can be targeted towards the surface receptors upon an external magnetic field. Once the field is removed, MNPs persist zero magnetism at room temperature resulting negligible agglomeration thereby preventing phagocytic uptake^8^. Due to this negligible aggregation, SPIONs will not elicit any adverse events that can lead to thrombosis. Heating efficacy of SPIONs, expressed in terms of specific absorption rate (SAR) is a pivotal factor determining the success of HT applications. SAR is solely related to the susceptibility loss mechanisms via either Néel relaxation and Brownian relaxation upon an AC magnetic field. If both processes take place concurrently, then the power dissipation is calculated by considering the relative contributions of both Neel and Brownian relaxation time denoted by τ_N_ and τ_B_ respectively. SAR value is consequently calculated in terms of the effective relaxation time, τ_effective_.

Suitable surface modification of SPIONs with biocompatible materials is crucial for achieving desired particle size in nanometer scale with enhanced saturation magnetisation (M_s_) and stable ferro fluids. These biocompatible surface coatings can render a protective layer on the surface of SPIONs for proper attachment to the surface receptors at the tumor sites. Coatings as surface active binding agents can attach to SPIONs surface via the end functional groups of the respective surfactants either through electrostatic or steric interactions. Surface modification of MNPs enhances steric and electrostatic repulsion between particles thereby avoiding self-aggregation and oxidation. This further assists in cellular uptake, stability, cell proliferation and viability, target specificity and prevents opsonisation when injected into the blood stream^9–12^.

Owing to the excellent magnetic properties, colloidal stability, biocompatibility and controlled size, magnetite (Fe_3_O_4_) NPs in the SPM size regime have been paid great attention^13^. In addition to these biomedical applications, Fe_3_O_4_ NPs are widely being investigated for sensing^14^ and energy^15^ domains. Magnetite NPs coated with surfactants should be in the desired size regime for easy penetration into the biological medium and also should permit excretion from the body by reticulo-endothelial system. NPs within 10–100 nm are considered as the preferential size regime^8^ for easy penetration which provides for a longer blood circulation time. Size of NPs is a crucial factor determining the half-life circulation time^16^. NPs with size < 10 nm are mostly cleared from the renal system whereas size > 200 nm size cause for phagocytic uptake, both being undesirable for biomedical applications^8^. Hence particle size is a key factor to be monitored during synthesis of NPs. Various hydrophilic biocompatible materials having distinct end functional groups such as –OH, –COOH, –NH_2_ etc. have been employed commonly for surface modifications^17^. Interaction between these coatings and surface atoms present on the magnetic core may develop a magnetically dead layer thereby diminishing magnetisation, a vital parameter to be focused in MHT applications. Therefore different magnetic response of SPIONs varies the heat generation rate and thereby the SAR value. SAR values can be varied due to the following reasons: (a) thickness of the surface coating on account of the fact that dense coatings hinder the Brownian motion of particles, (b) different hydrophilic nature of various surfactants and (c) different magnetisation possessed by the NPs due to the core and surfactant molecules. Since indirect contact between the core and the surrounding medium generates less heat, highly hydrophilic surfactants are preferred to provide direct contact resulting in higher SAR. For maximum SAR, the optimization of surface coating on MNPs based HT is an indispensable factor. For instance, highest SAR in MHT was achieved using an optimal coating of mPEG-2000 on Fe_3_O_4_ NPs^18^. Such optimized coated NPs have also exhibited excellent hyperthermic activity in both water and simulated body fluids. Similarly, PMA-ATA (pyromellitic acid-2-aminoterephthalic acid) dual surfactant coated SPIONs exhibited an enhanced SAR compared to the single chain surfactant coated ones^19^.

Though different studies investigated heat release in terms of various parameters such as particle size, saturation magnetisation, magnetic anisotropy, viscosity of the medium, and morphology^20–28^, a systematic study to understand how surface coatings tune the magnetic behaviour as well as the heat dissipation rate crucial for MHT studies is least experimented^29,30^. The motive of the present study is to investigate the effects of various surfactants on the structural, magnetic and thermal properties of SPM Fe_3_O_4_ NPs. We have synthesized surface functionalized SPM Fe_3_O_4_ NPs with six distinct surfactants such as glutamic acid (GA), citric acid (CA), poly ethylene glycol (PEG), polyvinylpyrrolidine (PVP), ethylene diamine (EDA) and cetyl-trimethyl ammonium bromide (CTAB) through co-precipitation method and studied towards MHT.

## Results

### Structural, microstructural and surface analysis

**Figure 1:**
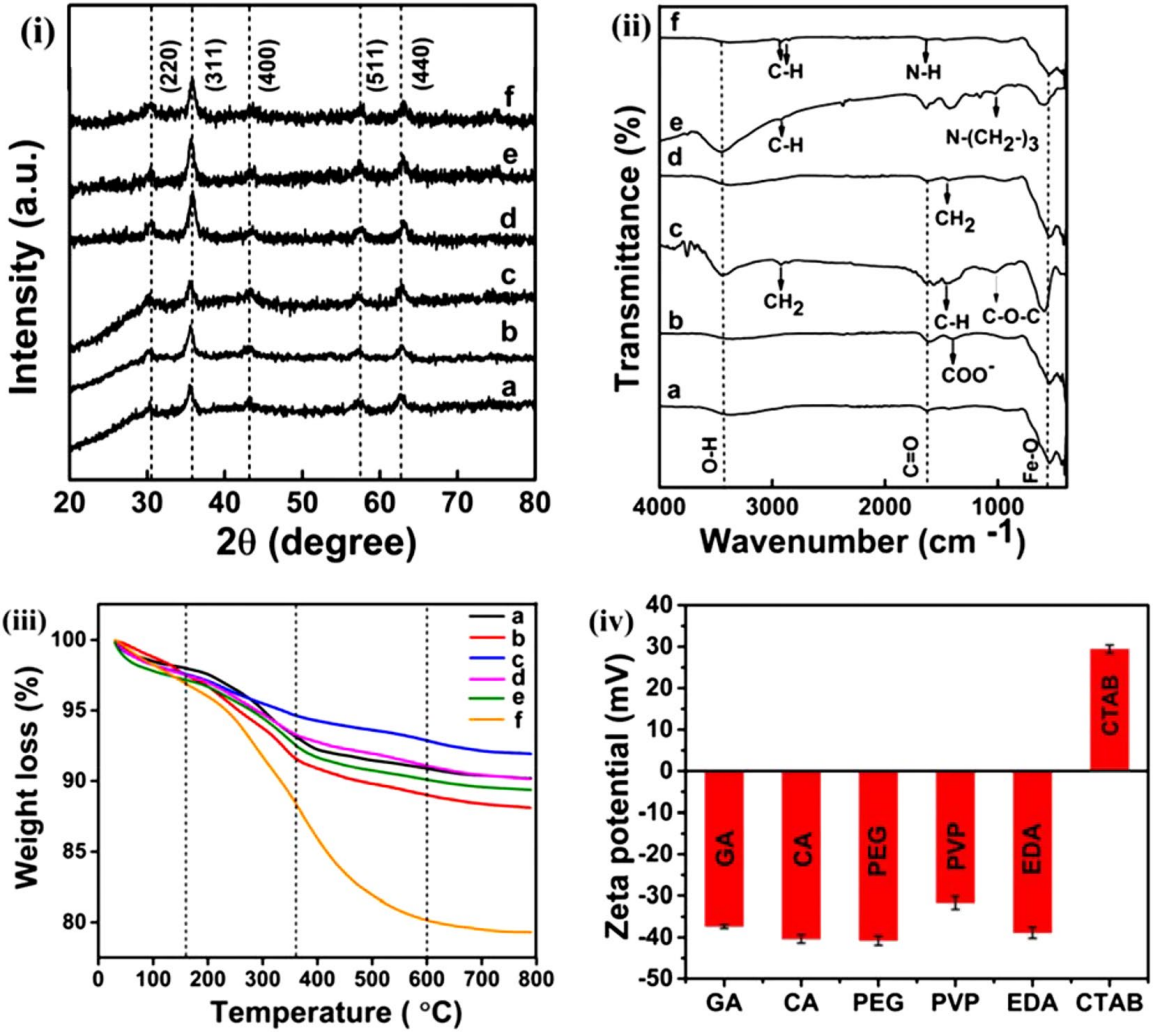
(**i**) XRD patterns, (**ii**) FTIR spectra, (**iii**) weight loss profile and (**iv**) zeta potential analysis. (a) GA-Fe_3_O_4_, (b) CA-Fe_3_O_4_, (c) PEG-Fe_3_O_4_, (d) PVP-Fe_3_O_4_, (e) EDA-Fe_3_O_4_ and (f) CTAB-Fe_3_O_4_ NPs.

XRD patterns of MNPs (Fig. 1i) with different surface functionalization display diffraction peaks at 2θ values of 30.4°, 35.5°, 43.2°, 57.1° and 63.1° corresponding to (220), (311), (400), (511) and (440) planes respectively indexed to cubic inverse spinel structure of Fe_3_O_4_ phase (JCPDS#19-0629). All the corresponding Bragg’s planes reveal the face-centred cubic structure with Fd-3m space group and lattice parameter in the range of 8.25–8.31 Å. Estimated average crystallite sizes are 8.1 ± 0.23, 8.4 ± 0.43, 8.8 ± 0.14, 8.3 ± 0.16, 9.5 ± 0.48 and 8.7 ± 0.28 nm for GA-Fe_3_O_4_, CA-Fe_3_O_4_, PEG-Fe_3_O_4_, PVP-Fe_3_O_4_, EDA-Fe_3_O_4_ and CTAB-Fe_3_O_4_ respectively. XRD pattern of the uncoated Fe_3_O_4_ (crystallite size ~ 12.36 ± 0.51 nm) is provided in Fig. S1 for comparison. The higher crystallite size in uncoated Fe_3_O_4_ ensures that functionalisation is necessary for controlling the size of NPs. FTIR spectra in Fig. 1ii show characteristic absorption bands at ~ 540 cm^−1^ corresponding to metal–oxygen vibrational band (Fe–O) at tetrahedral site^18^ and band near 3,436 cm^−1^ assigned to the hydroxyl (O–H) stretching vibrations of surface adsorbed water molecules. Lower intensity IR bands for GA-Fe_3_O_4_ at ~ 1,635 cm^-1^ and ~ 1,421 cm^−1^ correspond to the C=O stretching vibration of the COOH groups which ensures the binding of GA to the surface of Fe_3_O_4_ NPs by chemisorption of carboxylate ions^31^. Major characteristic broad band occurred at ~ 3,350 cm^−1^ attributes to the N–H stretching vibration of GA where it is overlapped by the O–H stretching. In case of CA–Fe_3_O_4_, IR band occurred at ~ 1,600 cm^−1^ confirming the C=O vibrations of the carboxylate groups of CA which binds with Fe atoms present on the surface. Band near 1,400 cm^−1^ coincides with COO^−^ symmetric stretching vibrations of COOH groups of CA^17^. From IR spectra of PEG-Fe_3_O_4,_ characteristic bands observed at ~ 2,924 cm^−1^, ~ 1,406 cm^−1^ and ~ 1,060 cm^−1^ correspond to –CH_2_, –CH and C–O–C ether bond stretching vibrations respectively of the PEG chains^32^. As an ethylene glycol derivative, the OH groups of the PEG result in the major intense peak in the range of 3,000–3,500 cm^−1^. Interaction via hydrogen bonding between the oxygen of PEG and the proton of protonated magnetite indicates PEG polymer chains were successfully grafted onto the Fe_3_O_4_ surface^33^. In the FTIR spectra of PVP coated magnetite, peaks observed at ~ 1,082 cm^−1^, ~ 1,420 cm^−1^ and ~ 1,645 cm^−1^ correspond to stretching vibrations of C–N stretching, CH_2_ and C=O respectively of PVP. Interaction between oxygen in carbonyl group of PVP and the proton present in the protonated magnetite via hydrogen bonding plays the PVP grafting on Fe_3_O_4_. In case of EDA coated magnetite, bands at ~ 1,022 cm^−1^ and ~ 2,930 cm^−1^ can be ascribed to the asymmetrical axial deformations in the group [N(–CH_2_–)_3_] of the tertiary amines and C–H axial deformities of the CH_2_ group present in EDA^34^. Band occurred at ~ 1,638 cm^−1^ may be due to the overlap of C=N and C=C bonds and at ~ 1,429 cm^−1^ attribute to the amide band. This indicates the amine group of EDA makes interaction with magnetite. CTAB coated magnetite spectra exhibits characteristic band at ~ 1,627 cm^−1^ of N–H stretching vibration of NH_2_ group and bands at ~ 2,848 cm^−1^ and ~ 2,918 cm^−1^ are attributed to CH vibration^35^. Antisymmetric vibration of C–N and CH_3_ stretching at ~ 960 cm^−1^ and ~ 1,470 cm^−1^ are also observed. These observed bands provide evidence for the interaction between the surface of magnetite and the chemisorbed CTAB molecules.

Weight loss profile as observed in TGA plots (Fig. 1iii) below 150 °C, between 150 and 380 °C and above 380 °C is correlated to the surface adsorbed water molecules/removal of hydroxyl group, the thermal decomposition of surfactants chemisorbed on the surface of NPs and thermally induced phase transformation of Fe_3_O_4_ to γ-Fe_2_O_3_, respectively. A total weight loss of around 10, 12, 8, 10, 11 and 21% was observed for GA, CA, PEG, PVP, EDA and CTAB coated Fe_3_O_4_ NPs respectively. Polymers exhibit an improved thermal stability compared to simple molecules or polysaccharides^36^ which keeps PEG more thermally stable than others. A higher weight loss for CTAB coated Fe_3_O_4_ compared to others indicates higher molecular mass of polymeric CTAB. Comparing the TGA plots, it is clear that the binding of surfactants on Fe_3_O_4_ was effectively achieved.

Zeta-potential (Fig. 1iv) showed that five out of six samples (GA-Fe_3_O_4_, CA-Fe_3_O_4_, PEG-Fe_3_O_4_, PVP-Fe_3_O_4_, EDA-Fe_3_O_4_) possess negative zeta potential (− 37.4, − 40.4, − 40.8, − 31.7, − 38.9 mV) and the remaining one (CTAB-Fe_3_O_4_) exhibited positive potential (+ 29.4 mV).

**Figure 2:**
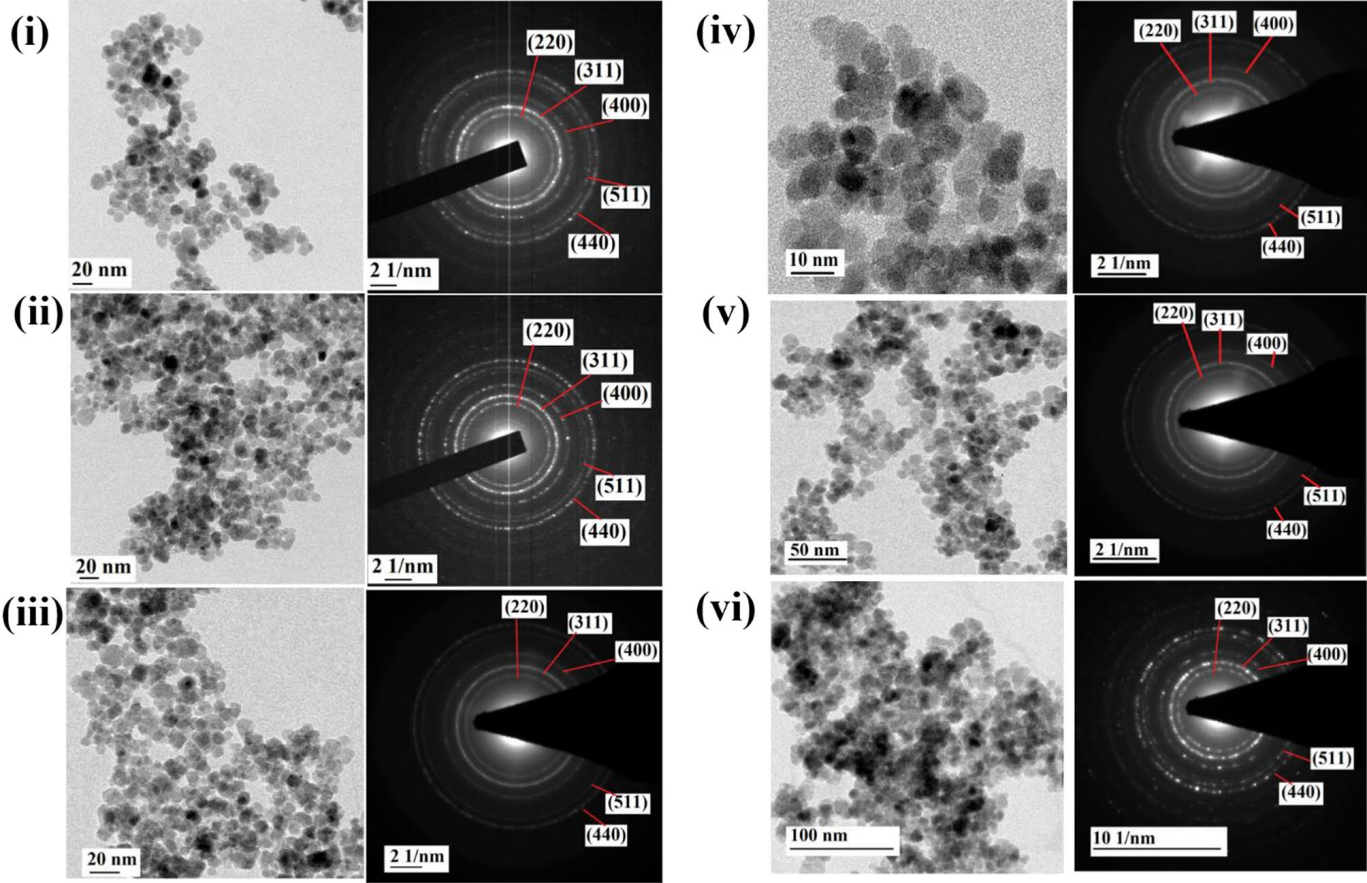
HRTEM images and SAED patterns. (**i**) GA-Fe_3_O_4_, (**ii**) CA-Fe_3_O_4_, (**iii**) PEG-Fe_3_O_4_, (**iv**) PVP-Fe_3_O_4_, (**v**) EDA-Fe_3_O_4_ and (**vi**) CTAB-Fe_3_O_4_ NPs.

TEM micrographs (Fig. 2) exhibit spherical and well resolved monodispersed particles. Effective surface modification and reduced magnetic dipolar interaction between the particles lead to this monodispersity. The average particle size was estimated using log normal distribution function (considering 50 particles using Image J software). The particles exhibited a narrow size distribution ranging from an average of 7.8 nm to 10.5 nm (provided in Fig. S2) for the characterised samples which correlates with crystallite size estimated from XRD analysis. Since the particle size is approximately similar to crystallite size, it can be inferred that a thin layer of coating of surfactants is present surrounding the iron oxide core^37^. In the case of GA-Fe_3_O_4,_ it is visible from Fig. S2i that the lattice fringes observed correspond to the (311) plane with an interplanar spacing of 2.5 Å and (400) plane with a spacing of 2.1 Å of magnetite. Moreover, one major crystal plane of GA could be identified with a lattice spacing of 3.82 Å (marked in the Fig. S2i). Selected area electron diffraction (SAED) patterns depicted in Fig. 2i match with XRD data which confirms the monocrystalline nature of cubic Fe_3_O_4_ NPs. Diffraction rings correspond to (220), (311), (400), (511) and (440) planes with corresponding interplanar spacing of 2.89 Å, 2.52 Å, 2.10 Å, 1.68 Å and 1.44 Å respectively of Fe_3_O_4_ (JCPDS card No. 19-0629). Further identification of surfactant (GA) was not detected from SAED pattern since the strongest line of GA might be present nearer to the central bright zone. SAED patterns of all other samples depicted in Fig. 2ii–vi exhibited similar electron diffraction patterns and matched the (hkl) indices of Fe_3_O_4_ based on calculated d-spacing.

**Figure 3:**
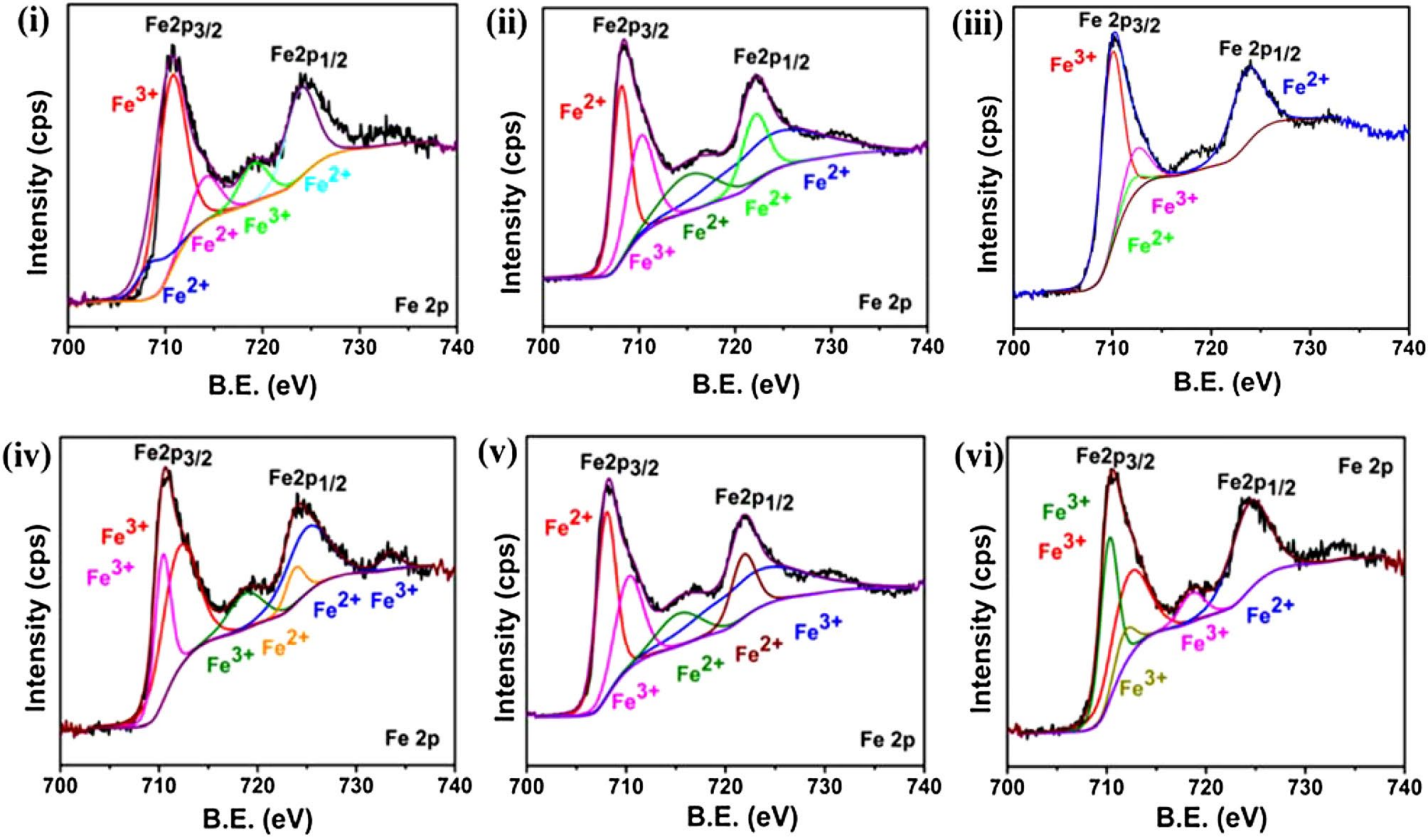
XPS analysis. Fe 2p XPS spectra of (**i**) GA-Fe_3_O_4_, (**ii**) CA-Fe_3_O_4_, (**iii**) PEG-Fe_3_O_4_, (**iv**) PVP-Fe_3_O_4_, (**v**) EDA-Fe_3_O_4_ and (**vi**) CTAB-Fe_3_O_4_ NPs.

Fe 2p XPS spectra are provided in Fig. 3 and analyses are summarised in supplementary material Fig. S3i–vi. The major peak at ~ 710 eV in Fe 2p spectra repeatedly observed (evident from Fig. 3i–vi) for GA-Fe_3_O_4_, CA-Fe_3_O_4_, PEG- Fe_3_O_4_, PVP-Fe_3_O_4_, EDA-Fe_3_O_4_ and CTAB-Fe_3_O_4_ NPs confirms the presence of magnetite phase^38^. In the deconvoluted Fe2p spectrum of GA-Fe_3_O_4_ (Fig. 3i), five peaks are observed at 723.9, 719.7, 714.1, 710.8 and 708.7 eV^39^. Major peak at 710.8 eV confirms the characteristic peak from Fe2p_3/2_ core level electrons attributing to the Fe^3+^ octahedral site^40^. Peak at 723.9 eV confirms Fe^2+^ species in the octahedral sites which attributes to the carboxylate-iron bond. A slight chemical shift to the higher binding energy occurred for 710.8 eV and 723.9 eV (deviation of 0.8 eV and 0.9 eV respectively) from that of metallic Fe^41^. This shift may be due to the bonding of Fe with O ions or due to Fe and COO^−^. Fe^2+^ species are found at binding energy of 708.7 eV, 714.1 eV and Fe^3+^ species in the tetrahedral site exhibit a weak shoulder at 719.7 eV corresponds to the Fe2p_3/2_ of Fe_3_O_4_. Fundamentally, Fe^2+^ ions determine the magnetic moment in the lattice. XPS profile of O 1s electrons in GA-Fe_3_O_4_ (provided in S3i) deconvolute into three peaks located at 529.91, 531.03, 531.84 eV corresponds to the oxygen species in Fe–O, C–O and C=O respectively^42^. Also from C 1s XPS spectrum, two peaks located at 284.76 eV and 288.5 eV are assigned to the carbon atom (either bound to carbon or to hydrogen) and carbon atom in COOH or COO^-^ peak respectively^38^. These results suggest that the surface modification with GA on Fe_3_O_4_ NPs surfaces have accomplished successfully. Similarly, XPS analysis given in Fig. S3ii–vi for other samples inferred that the position of Fe^2+^ and Fe^3+^ peaks in Fe 2p region could vary based on the ligand species and also confirms the successful synthesis of corresponding surfactant coated Fe_3_O_4_ NPs. Table S1 provides a summary of XPS binding energies and shifts for all coated samples.

### Magnetic properties

**Figure 4:**
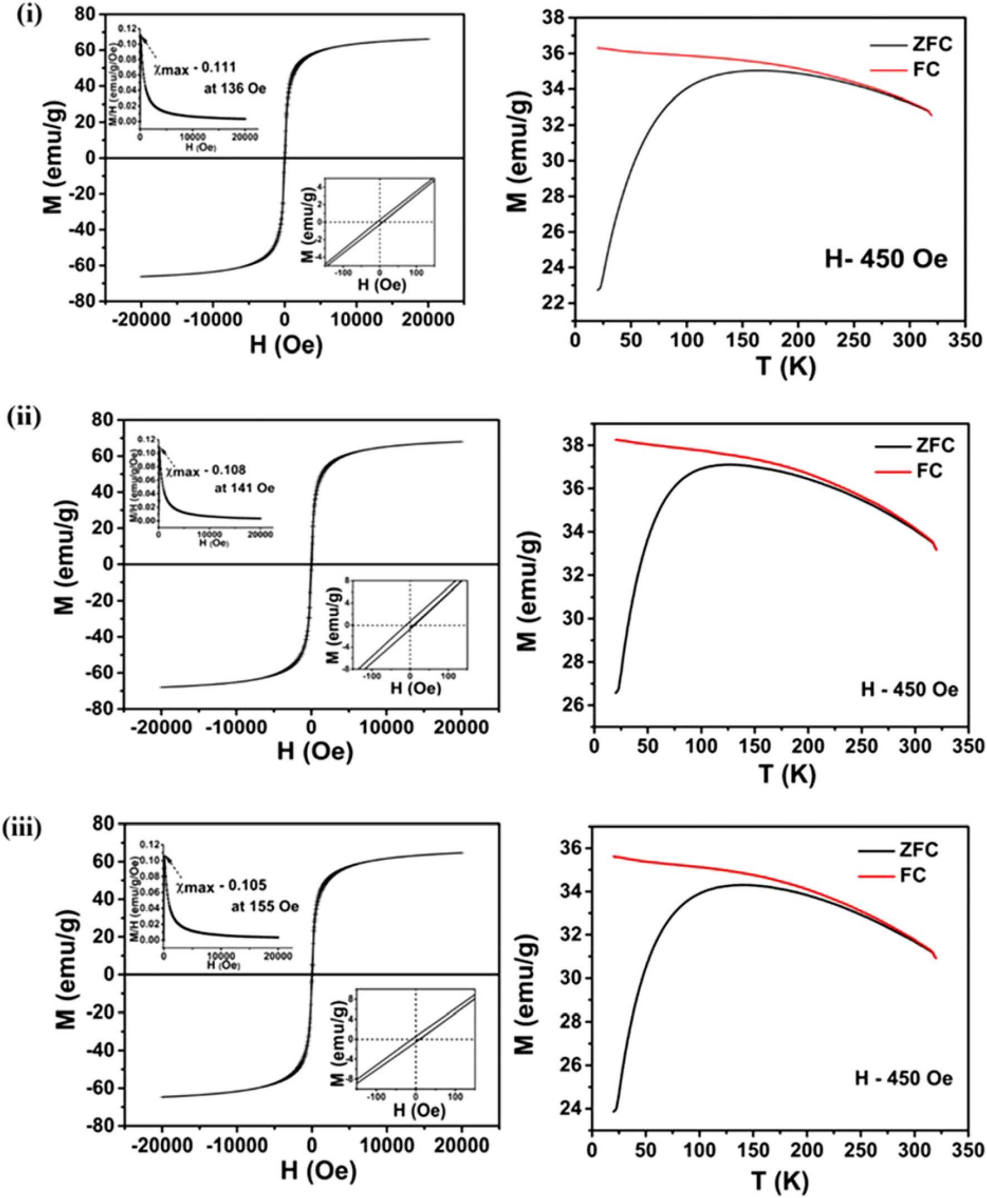

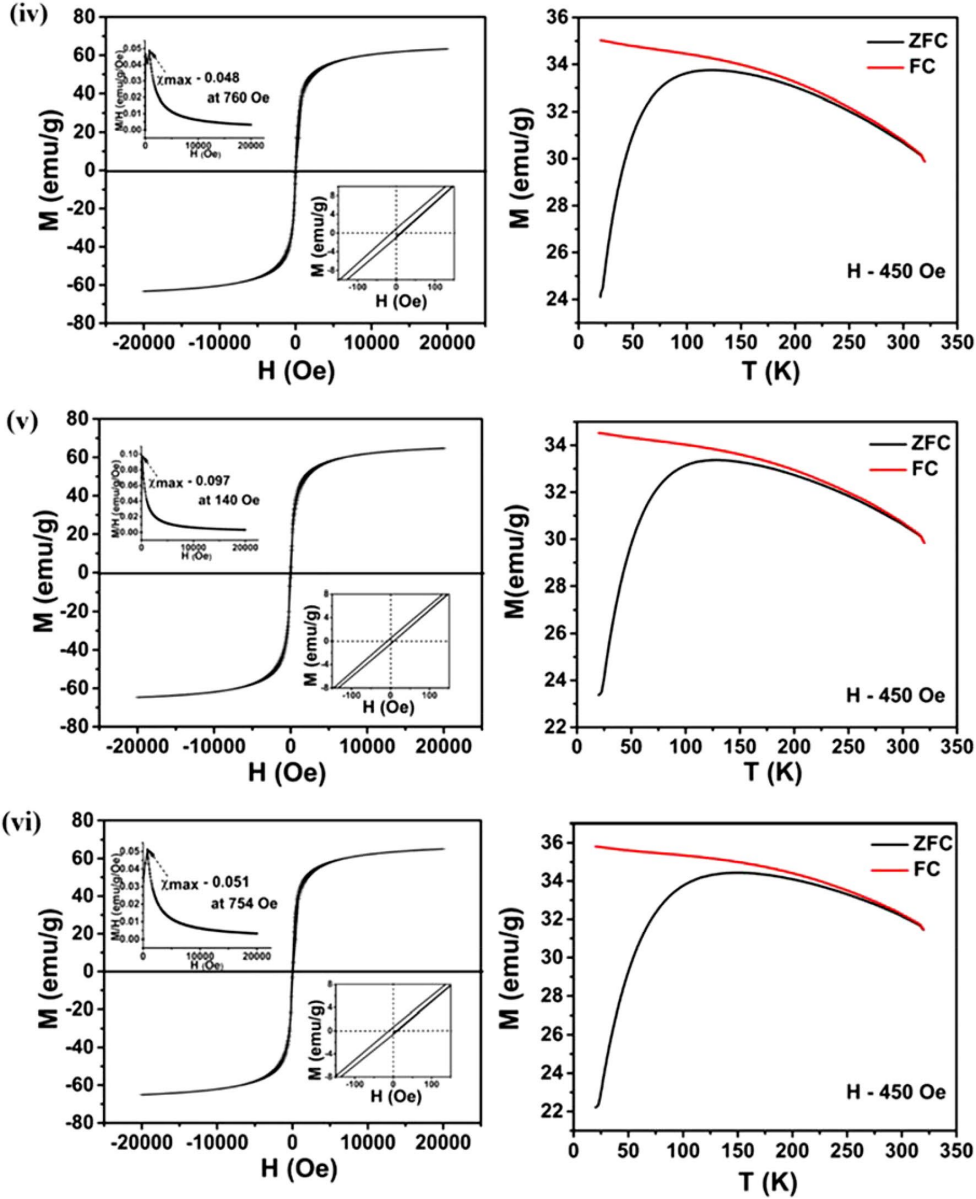
Superparamagnetic behaviour exhibited by surface functionalised samples. M-H curves at 300 K, maximum magnetic susceptibility, χ_max_ at 300 K (insets), coercivity (H_c_) (insets) and M-T plots of (**i**) GA-Fe_3_O_4_, (**ii**) CA-Fe_3_O_4_, (**iii**) PEG-Fe_3_O_4_, (**iv**) PVP-Fe_3_O_4_, (**v**) EDA-Fe_3_O_4_ and (**vi**) CTAB-Fe_3_O_4_ NPs.

Magnetic measurements confirm the SPM nature for all samples, as can be seen in Fig. 4i–vi. Saturation magnetisation (M_s_) values of GA, CA, PEG, PVP, EDA and CTAB coated samples are found to be 66, 68, 65, 63, 65 and 65 emu/g respectively at applied magnetic field of 2 kOe. In addition, hysteresis exhibits negligible coercivity (~ 10 Oe) for all the samples (shown in inset). Among those, CA-Fe_3_O_4_ exhibits higher magnetisation whereas PVP-Fe_3_O_4_ exhibits the least. A slight presence of maghemite in PVP-Fe_3_O_4_ causes for the lowest magnetisation which is clearly evident from XPS analysis (given in Supplementary Material S3iv). Magnetic measurement exhibiting SPM nature for the uncoated Fe_3_O_4_ is provided in Fig. S4. M_s_ value of the uncoated Fe_3_O_4_ NPs is observed to be 73 emu/g which is higher than the coated samples. Though surfactant coated MNPs are more free and prone to rapid alignment with external field compared to the uncoated ones^43^, the effective magnetic moment of such NPs is observed to be lower than the bare ones. Magnetic moment values (µ) are estimated to be 2.76, 2.84, 2.71, 2.63, 2.71 and 2.71 µ_B_/Fe_3_O_4_ respectively for GA, CA, PEG, PVP, EDA and CTAB coated samples whereas it is found to be 3.07 µ_B_/Fe_3_O_4_ for bare one. Decrement in magnetic moment (compared to bulk value) is attributed to the non-collinear spin structure originated from the surface spin pinning and coatings at the interface of NPs. As the measured magnetic moment is less than theoretical value (4 µ_B_) and the diameter of the particles obtained from TEM being much less than the single domain critical diameter (80 nm, reported for magnetite)^10,44^, the synthesized NPs are considered to be single domain. Moreover, from M-H curve it is observed that NPs exhibit maximum susceptibility (χ_max_ = dM/dH) at low applied field. The magnetic susceptibility for all the NPs at room temperature is shown in Fig. 4 (inset), [$\upchi = \frac{M(H,T)}{H}$]. At room temperature, the maximum value of susceptibility (reported in Table 1) is found at fields of 136 Oe, 141 Oe, 155 Oe, 760 Oe, 140 Oe and 754 Oe respectively for the GA, CA, PEG, PVP, EDA and CTAB coated samples.

**Table 1:** Magnetic properties such as saturation magnetization (M_s_), magnetic moment (m), anisotropy energy constant (*K*) and susceptibility (χ) values.

Sample	Saturation magnetization (M_s_) (emu/g)	Magnetic moment (m) (µ_B_)	Anisotropy energy constant (*K*) (× 10^5^ erg/cm^3^)	Magnetic susceptibility (χ) (emu/gOe) for 450 Oe at 300 K	Maximum magnetic susceptibility (χ_max_) at 300 K (emu/gOe) and corresponding fields
GA-Fe_3_O_4_	66	2.76	2.05	0.074	0.111 at 136 Oe
CA-Fe_3_O_4_	68	2.84	1.76	0.072	0.108 at 141 Oe
PEG-Fe_3_O_4_	65	2.71	1.68	0.069	0.105 at 155 Oe
PVP-Fe_3_O_4_	63	2.63	1.72	0.042	0.048 at 760 Oe
EDA-Fe_3_O_4_	65	2.71	1.21	0.068	0.097 at 140 Oe
CTAB-Fe_3_O_4_	65	2.71	1.33	0.044	0.051 at 754 Oe

Zero field cooled (ZFC)/field cooled (FC) curves at an applied field of 450 Oe are depicted in Fig. 4i–vi. ZFC curve displays an increment in magnetisation with temperature and reached the maximum corresponding to the blocking temperature, T_B_. Below T_B_, the particles are in blocked state exhibiting an irreversible magnetisation with ferromagnetic nature whereas above T_B_, the particles are in a reversible SPM nature. Increment in magnetisation in ZFC curve originates from the contribution of blocked magnetic moments. Similarly, all the FC magnetisation decreases with rise in temperature which may be due to the randomisation of blocked magnetic moments upon thermal energy^45^. Such decrement in magnetisation below T_B_ confirms the SPM nature. Similar magnetic behaviour reflects similar size and composition of the magnetic cores of all samples. Calculated T_B_ values for the maximum magnetisation are tabulated in Table S2. T_B_ values depend on the effective anisotropy and particle size according to^46,47^: $K=25 \times \frac{{k}_{B}{T}_{B}}{V}$ where *K* is the effective anisotropy energy constant, *k*_*B*_ is the Boltzmann constant, *T*_*B*_ is the blocking temperature at which thermal energy becomes comparable to magnetic anisotropy energy barrier where the particle goes into the SPM regime and *V* is the volume of the MNP based on the particle size determined from HRTEM. The calculated *K* values for GA-Fe_3_O_4_, CA-Fe_3_O_4_, PEG-Fe_3_O_4_, PVP-Fe_3_O_4_, EDA-Fe_3_O_4_ and CTAB-Fe_3_O_4_ are given in Table S2. It is found that GA-Fe_3_O_4_ exhibits comparatively enhanced effective anisotropy of 2.05 × 10^5^ erg/cm^3^ than others which may contribute for higher heating efficiency under AC magnetic field.

### Calorimetric heating performance

**Figure 5:**
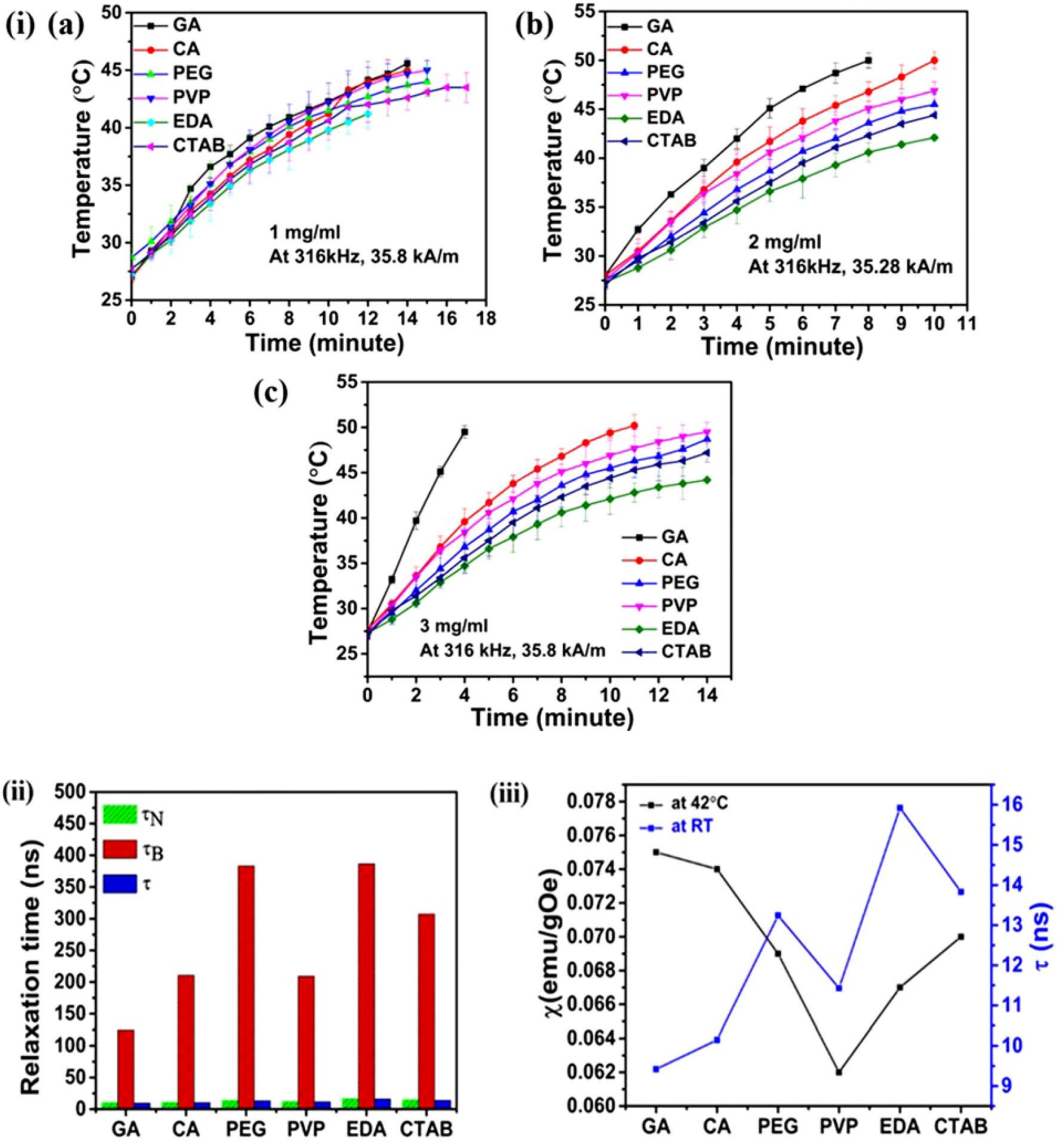
(**i**) Temperature–time profiles for the colloidal dispersions of GA, CA, PEG, PVP, EDA and CTAB coated Fe_3_O_4_ for concentrations (a) 1 mg/ml, (b) 2 mg/ml and (c) 3 mg/ml under alternating magnetic field, (**ii**) relaxation time graph and (**iii**) graph between magnetic susceptibility at 42 °C (HT monitoring temperature) measured at 450 Oe (applied magnetic field) and effective relaxation time at RT.

In order to assess the heating efficacy of the samples, temperature–time calorimetric evaluations under magnetic field parameters of 450 Oe and 316 kHz were conducted. In biomedical applications, generally surface modified NPs are used to conjugate drug or biological molecules in order to target tumor specific surface receptors, however, for a comparative study, heating efficacy of uncoated NPs is also analysed and provided in Fig. S5. In view of clinical aspects for effective MHT applications, a minimal concentration range^48,49^ (1 mg/ml, 2 mg/ml and 3 mg/ml) was selected for present study. The selected concentration can produce significant heating within 10 min without reaching the boiling point of water. An in vivo study experimented on prostate cancer cells have chosen a concentration of 41.7 mg/ml^50^. Similarly, a successful in vivo MHT in animal models led to the first human clinical trial by Johannsen et al. for the treatment of prostate cancer^51^ have chosen a concentration of 120 mg/ml. As evident from Fig. 5i(a,b) a gradual rise in temperature with time was observed for all the samples. Time required to attain the required HT temperature (42–46 °C) varies with different NPs under identical experimental conditions. Moreover, Fig. 5i(a,b) demonstrated that heating response is concentration dependent for all the samples. 1 mg/ml samples attained a peak value at ~ 43 °C and became saturated afterwards as generation of heat is balanced by dissipation of heat in MNPs. However, in the case of 2 mg/ml concentration, all samples reached beyond the HT limit (~ 46 °C). This rise in temperature can be a consequence of increased particle concentration in the solvent. In terms of time duration to attain clinically relevant HT response under magnetic field exposure, 1 mg/ml NPs is found to reach 43 °C after a prolonged exposure (~ 10 min) whereas in the case of 2 mg/ml NPs, HT limit (43 °C) was obtained within a comparatively lesser time span (~ 5 min). In the current context, it can be concluded that higher concentrated samples (2 mg/ml) increases the heating efficacy within shorter duration. HT ability is in the order of GA-Fe_3_O_4_ > CA-Fe_3_O_4_ > PVP-Fe_3_O_4_ > PEG-Fe_3_O_4_ > CTAB-Fe_3_O_4_ > EDA-Fe_3_O_4_ for 2 mg/ml. Moreover, relaxation time graph in Fig. 5ii illustrates the contribution of magnetic moment relaxation of NPs. SAR was calculated (in Table 2) from the initial slope of temperature–time curves as the temperature response with time is non-linear for non-adiabatic systems (due to thermal loss). The SAR of uncoated samples (depicted in Fig. S5) at NPs concentrations of 1 mg/ml, 2 mg/ml and 3 mg/ml are found to be higher than the coated samples due to the higher magnetisation obtained. An increment in SAR upon increasing the concentration can also be observed in the uncoated sample. Highest SAR values are recorded for GA-Fe_3_O_4_ NPs whereas it is lowest for EDA-Fe_3_O_4_ NPs. Moreover, when concentration of GA-Fe_3_O_4_ NPs increases from 1 to 2 mg/ml, SAR has increased by 91%. It can be perceived that an increment in concentration of NPs is associated with a corresponding enhancement of SAR value. Highest SAR of 115 W/g achieved for GA-Fe_3_O_4_ NPs is the one which possesses the higher magnetic anisotropy with faster relaxation time (calculated in the previous section) compared to others. Research study conducted by Hergt et al. demonstrated that the heat dissipation of MNPs possessing an SAR value of magnitude 100 W/g is considered to be a pertinent option for MHT applications^52^. Even though M_s_ value obtained for CA-Fe_3_O_4_ is highest among all, faster relaxation time for magnetic flipping obtained for GA-Fe_3_O_4_ has led to the maximum SAR value. Similar to the above case, PVP coated MNPs possess a lower M_s_ value of 63 emu/g compared to PEG, EDA and CTAB coated MNPs (similar M_s_ of 65 emu/g), however, due to a comparatively higher anisotropy and faster relaxation time resulting in a higher SAR. SAR value remains lowest in case of EDA-Fe_3_O_4_. In addition to the relaxation mechanisms, the magnetic susceptibility at HT temperature (42 °C) for the applied field (450 Oe) is also plotted (data obtained from M-T curve) and shown in Fig. 5iii.

**Table 2:** Magnetic parameters, SAR and ILP of different samples.

Sample	Relaxation time (ns)			Magnetic susceptibility (χ) (emu/gOe) for HT applied field (450 Oe) at 42 °C	SAR (W/g)			ILP (nHm^2^/kg)		
	${\uptau }_{\mathrm{N}}$	${\uptau }_{\mathrm{B}}$	$\uptau$		1 mg/ml	2 mg/ml	3 mg/ml	1 mg/ml	2 mg/ml	3 mg/ml
GA-Fe_3_O_4_	10.19	124.9	9.42	0.075	60	115	130	0.15	0.3	0.34
CA-Fe_3_O_4_	10.66	210.6	10.15	0.074	48	97	67	0.12	0.23	0.17
PEG-Fe_3_O_4_	13.71	383	13.24	0.069	49	80	53	0.13	0.17	0.14
PVP-Fe_3_O_4_	12.09	209.2	11.43	0.062	56	92	63	0.14	0.19	0.16
EDA-Fe_3_O_4_	16.61	386.3	15.92	0.067	44	66	44	0.11	0.14	0.11
CTAB-Fe_3_O_4_	14.48	307.4	13.83	0.070	47	71	47	0.12	0.16	0.12

**Figure 6:**
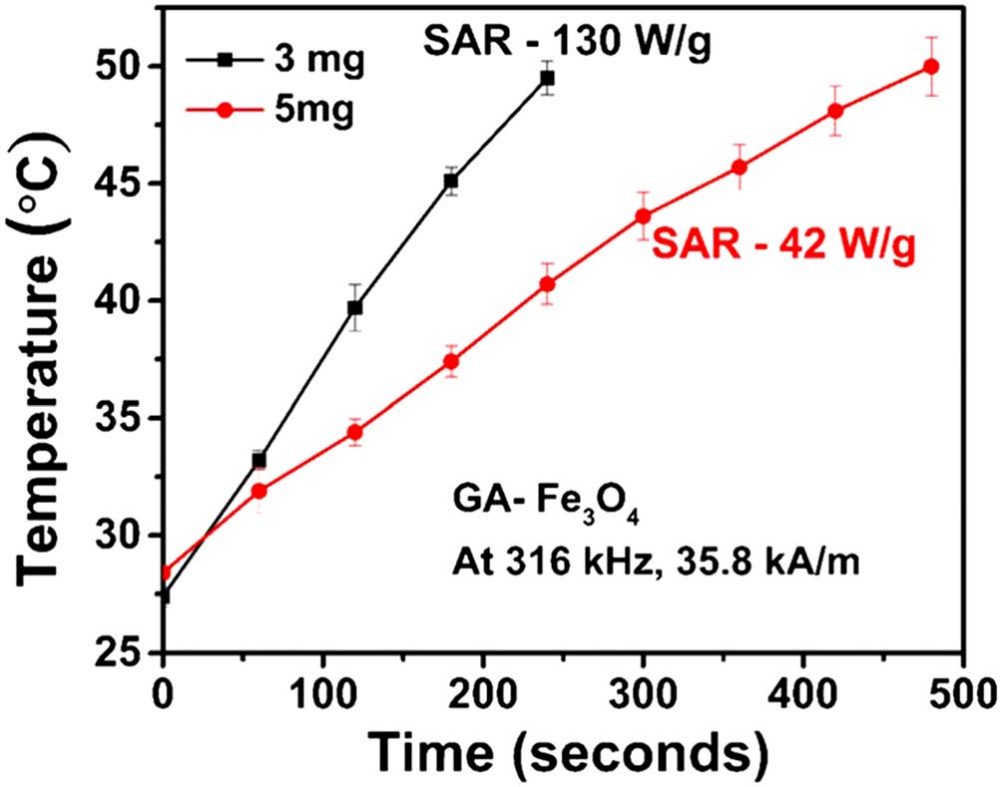
Temperature–time profiles for the colloidal dispersions of GA coated Fe_3_O_4_ for higher concentrations showing its heating saturation ability.

For elucidating the optimal concentration of all MNPs on heating efficiency, all the samples were further subjected to HT at a comparatively higher concentration of 3 mg/ml (Fig. 5i(c)) and SAR was determined (Table 2). Highest SAR of 130 W/g was obtained for GA-Fe_3_O_4_ whereas SAR values for the remaining samples exhibited a demoting trend with SAR values similar to 1 mg/ml concentrated samples. Since heat dissipation due to magnetic moment flip under AC magnetic field still persists leading to a higher magnitude of SAR value for 3 mg/ml GA-Fe_3_O_4_, optimum concentration for heating ability needs to be identified. Accordingly, further analysis of HT studies for 3 mg/ml and 5 mg/ml were performed (Fig. 6) and SAR values were determined. A substantial decrease in SAR of 42 W/g has been determined for 5 mg/ml concentration due to the presence of strong demagnetising interactions between the NPs.

## Discussion

The present work has investigated the heating potentiality of Fe_3_O_4_ NPs coated with various surfactants such as GA, CA, PEG, PVP, EDA and CTAB by elucidating the influence of surfactants on the magnetic as well as inductive thermal properties of Fe_3_O_4_ NPs. Well defined broad peaks obtained from XRD revealed the nano-crystalline nature of Fe_3_O_4_ and absence of any impurity peaks confirmed the single-phase nature. These various surfactant-coated Fe_3_O_4_ NPs neither exhibited phase transitions nor phase shifts suggesting that introduction of surfactants during the reaction does not affect the crystal structure. This is evident from the XRD patterns of both coated and uncoated Fe_3_O_4_ NPs exhibiting same peaks. Reduced crystallite sizes obtained (8–10 nm) after surface modification is attributed to the different nature and charge of surfactants used^32^. Hence it is clear that surfactants influence the crystallite size rather than its structure. From the FTIR data, metal–oxygen band commonly present at ~ 540 cm^−1^ confirms the presence of Fe^3+^ ions which gets coordinated through the corresponding functional group. Though Fe^2+^ ions are present in octahedral sites, Fe^3+^ ions in the tetrahedral site at the periphery have a strong preference for the feasible coordination. This inference is supported by the results of TGA, XPS, zeta potential and the proposed reaction mechanism.

All the coated samples are highly stable due to significant interparticle electrostatic or steric repulsive force and consequently good dispersion stability as indicated by zeta potential results. Existence of negative charge on magnetite surface is ascribed to the surface deprotonation and presence of hydroxyl, carbonyl or carboxylic groups attachment^53^ whereas positive charge is owing to the cationic surfactant used. GA (with two carboxyl and one amino group) and CA (with three carboxyl and one OH group) are polysaccharides possessing strong binding affinity to Fe_3_O_4_^36^. For both cases, chemisorption occurs by the coordination of COOH group with the Fe–OH molecules on the surface of Fe_3_O_4_^54^. Similarly, PEG having hydroxyl groups and PVP having carbonyl group through hydrogen bonding are responsible for binding interactions with the surface of Fe_3_O_4_ NPs^53,55^. EDA, a bi-dentate ligand with two nitrogen atoms at the centre having lone pair serve as Lewis base which coordinates with the surface of Fe_3_O_4_^56^. CTAB provides a structure of 16-carbon and an ammonium group which acts as a long tail and head which is attached to three methyl groups^57^. Hence, the presence of quaternary amine at the outermost layer in CTAB is responsible for the binding interaction and positive charge^58^. More suitably, electrostatic stabilisation is obtained in ionic surfactant like CA, GA and EDA whereas steric stabilisation dominates in polymeric surfactants like PEG, PVP and CTAB.

The possible reaction mechanism leading to the formation of magnetite (Fe_3_O_4_) from the Fe- precursors such as ferrous (FeCl_2_) and ferric chloride (FeCl_3_) with alkali (NH_4_OH) precipitation may be given as^59^:

$${\mathrm{FeCl}}_{3}+{3\mathrm{NH}}_{4}\mathrm{OH}+{\mathrm{H}}_{2}\mathrm{O}\to {\mathrm{Fe}(\mathrm{OH})}_{3}+3{\mathrm{NH}}_{4}\mathrm{Cl}+{\mathrm{H}}_{2}\mathrm{O}$$

$${\mathrm{FeCl}}_{2}+{2\mathrm{NH}}_{4}\mathrm{OH}+{\mathrm{H}}_{2}\mathrm{O}\to {\mathrm{Fe}\left(\mathrm{OH}\right)}_{2}+2{\mathrm{NH}}_{4}\mathrm{Cl}+{\mathrm{H}}_{2}\mathrm{O}$$

$${\mathrm{Fe}(\mathrm{OH})}_{2}+{2\mathrm{Fe}(\mathrm{OH})}_{3}\stackrel{\Delta }{\to }{\mathrm{Fe}}_{3}{\mathrm{O}}_{4}+{4\mathrm{H}}_{2}\mathrm{O}$$

Alkaline NH_4_OH plays a crucial role for co-precipitation of Fe^2+^ and Fe^3+^ ions present in the mixed solution by providing hydroxyl (OH^−^) ions to precipitate unstable metallic hydroxides. During heating, water (H_2_O) molecules get eliminated from the metal mixed hydroxides leading to the formation of Fe_3_O_4_. Inert atmosphere (nitrogen) used throughout the experiment prevented oxidation and helped in size reduction of NPs when compared with other methods without removing oxygen^43,60^. The proposed mechanisms are illustrated in Fig. S6. It is inferred that –COOH groups present in GA and CA linked with the –OH groups available on the surface of Fe_3_O_4_ as COOH groups possess a higher affinity towards metallic oxides. It was reported that MNPs synthesized via co-precipitation method comprised of a number of OH groups on the surface^61,62^ which can bind surface active agents. In the present study, Fe_3_O_4_ NPs in aqueous medium resulted in water dissociation to produce OH groups on the surface. Hence, stabilisation of Fe_3_O_4_ NPs has been achieved by the direct attachment of the respective surfactants (containing carboxyl, hydroxyl or amino groups) to the OH group present on the surface of the precipitated NPs. Here, Fe–OH bond on magnetite NPs surface reacts with COOH group present in both GA and CA molecule through an acid–base reaction resulting Fe–O–C species by eliminating H_2_O. It is reported that COOH or OH groups act as hydrogen donor or acceptor with the Fe_3_O_4_ NPs via hydrogen bond^60^. OH and C=O groups present in PEG^63^ and PVP respectively are responsible for interaction via hydrogen-bonding interactions. Positively charged hydrogen ion (H^+^) from NH_2_ groups in EDA reacts with OH^-^ leaving NH^−^ which coordinates with Fe^2+^ ions. Similarly, positively charged nitrogen (N^+^) group present in CTAB is responsible for interaction. These conclusions strongly support the surface modification of Fe_3_O_4_ NPs with varying surfactants.

Even though all MNPs have exhibited SPM nature experimentally, negligible coercivity occurred due to difference in proportion of factors such as particle volume, magnetic anisotropy and thermal energy. These factors concomitantly influence the magnetic moments to randomly fluctuate resulting in SPM behaviour. Though significant size effect on magnetic saturation values for various surfactant coated NPs was not observed, observed M_s_ values may be attributed to the varying surface state of NPs. Surfactants co-ordinately anchored to the Fe_3_O_4_ surface may alter the surface state thereby varying the magnetic properties^64^. Each surfactant has interacted with the magnetic core differently exhibiting varying magnetic response. Coatings affect the canting angles of magnetic moments of Fe core in its magnetic sublattice generating magnetic spin disorder^65^ which results in lower magnetisation than the bulk magnetite value^35^. This is more evident from the higher M_s_ value obtained for the uncoated Fe_3_O_4_ NPs i.e., magnetisation value of Fe_3_O_4_ NPs reduces after surface functionalization. Consequently, the obtained saturation magnetization values arise as a result of both the volumes of respective diamagnetic coating and total iron oxide. Similarly, substantial number of Fe^2+^ ions present in GA, CA, PEG, and EDA coated samples are responsible for enhanced magnetic susceptibility compared to PVP and CTAB coated ones which is evident from the XPS spectra (details given in Table S1). Moreover, different anisotropy (*K*) values obtained (Table S2) imply the different amount of energy required to orient the entire magnetic moment of corresponding grains (iron oxide core and coatings).

Since all the samples have approximately similar size and magnetisation values, variation in hyperthermic ability can be attributed to the distinctive Neel–Brownian relaxation mechanisms induced by different coatings^18^. This eventually affects the heat dissipation upon an external magnetic field. Magnetic anisotropy is a predominant factor affecting the Neel-Brownian relaxation of MNPs subjected to an AC magnetic field^66^. When magnetic spin reversal and particle rotation occurs in parallel, the effective relaxation time is calculated using the equation: $\frac{1}{{\varvec{\uptau}}}=\frac{1}{{{\varvec{\uptau}}}_{\mathbf{N}}}+\frac{1}{{{\varvec{\uptau}}}_{\mathbf{B}}}.$ In the present study ${\uptau }_{\mathrm{N}}$ and ${\uptau }_{\mathrm{B}}$ are calculated using the modified Neel relaxation and Brownian equations^18^, as can be seen in Table 2. Though Brownian mechanism is not directly linked to magnetic behaviour associated with MNPs, it holds a noteworthy impact to intensify the magnetic behaviour in terms of increment in NP size within the SPM limit. In this study, Brownian contribution is negligible compared to Neel contribution which is evident from the calculated values. Therefore, utmost contribution to heat dissipation is attributable to the Neel losses and its relaxation time, τ_N_ is found to be effectively controlled by the anisotropy constant *K*.

Magnetic field reversal time of 5.03 × 10^–7^ s (τ_m__=_ $\frac{1}{2\mathrm{\pi \upsilon }}$) is found to be shorter than the magnetic relaxation time (τ) (obtained in Table 2) of NPs on exposure to magnetic field of 316 kHz frequency which strongly confirms the heat dissipation is strictly via magnetic moment relaxation mechanisms. Faster relaxation time, which denotes maximum number of sufficient attempts to overcome the energy barrier for magnetisation reversal, occurs for GA-Fe_3_O_4_ NPs. Even though M_s_ value obtained for CA-Fe_3_O_4_ is highest among all, faster relaxation time for magnetic flipping obtained for GA-Fe_3_O_4_ has led to the maximum SAR value. Even though PVP coated NPs possess a lower M_s_ than PEG, EDA and CTAB, it results in a higher SAR due to a comparatively higher anisotropy and faster relaxation time. A maximum magnetic susceptibility is observed for GA-Fe_3_O_4_ at 42 °C among all samples. In our investigation, anisotropy, magnetic susceptibility and effective relaxation time dominantly contributed to SAR value. In addition to this, SAR is found to be reduced for higher concentrated samples due to increased magnetostatic interactions (lowers the anisotropy energy barrier). Such interactions consequently lead to the reduction in Neel-Brownian relaxation (no mobility between NPs)^21^ i.e., NPs not at all rotate under the applied magnetic field as they are considered as large entities possessing weak remnant magnetization so the torque undergone is trivial. It can also be concluded that magnetic field lines closure configuration reduces the interaction between each NP thereby causing for reduced magnetic moment of aggregates.

**Figure 7:**
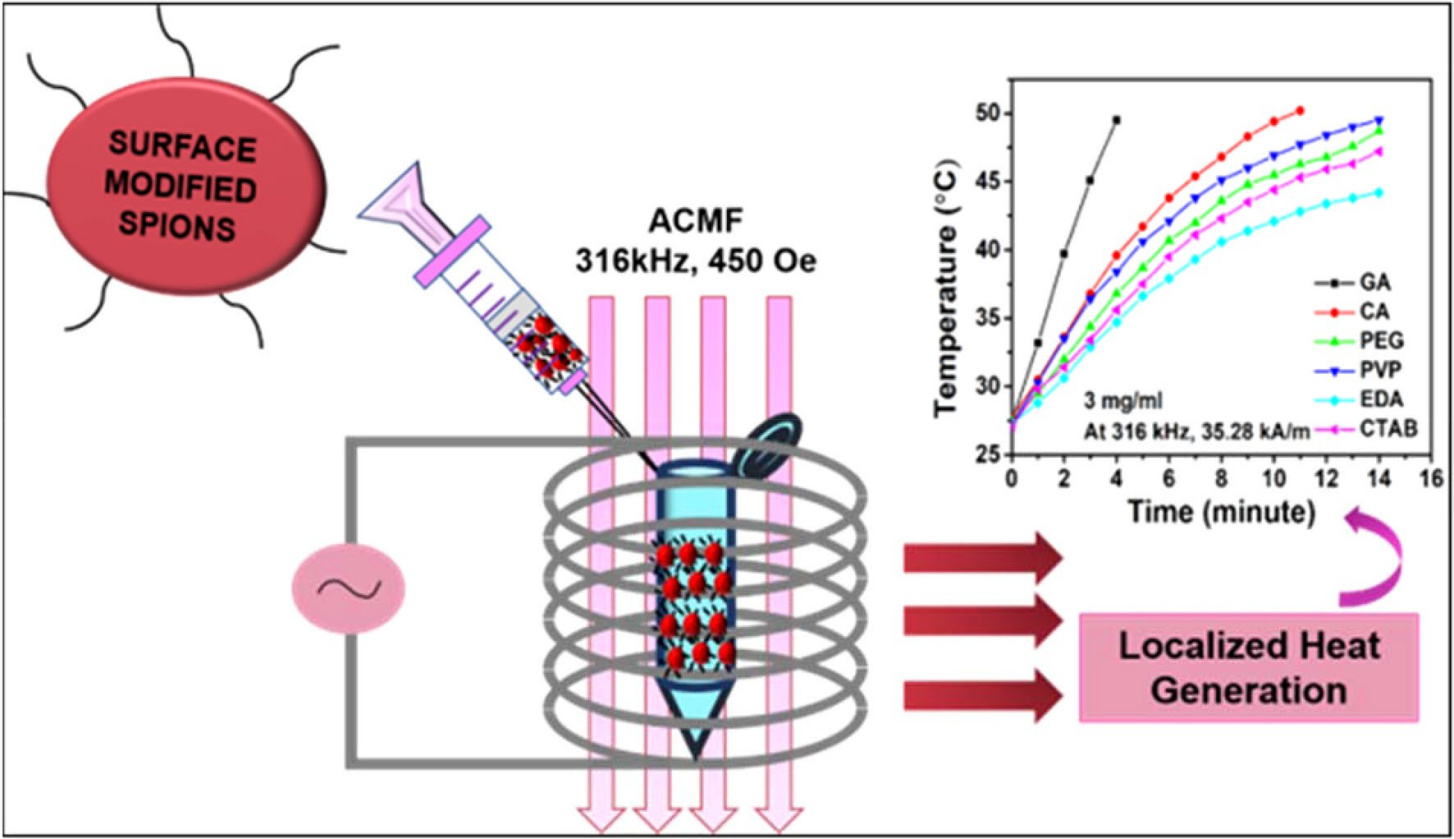
Schematic illustration of calorimetric heating performance mediated by colloidal SPIONs.

Specifically, the study revealed that GA coated Fe_3_O_4_ NPs with enhanced anisotropy, enhanced magnetic susceptibility, faster relaxation time and with a higher SAR release a larger amount of heat which is potentially stable for clinical MHT applications compared to other samples. Moreover, the intrinsic loss power (ILP) values of the corresponding surfactant coated Fe_3_O_4_ NPs were calculated and summarised in Table 2. Though the calculated ILP values are lower, these MNPs can still be used as nano heaters for MHT therapy since these values are similar to certain commercially available functionalised superparamagnetic ferrofluids such as Nanomag-D-spio (0.23 nHm^2^/kg), Micromode ferrofluids (0.15–0.35 nHm^2^/kg) etc^67^. Further the functionalised NPs are biocompatible in nature and can be conjugated with other drug molecules for chemothermal therapy of cancer as evident from our earlier published results^26,27,68^. Figure 7 shows the schematic illustration of as synthesized surface modified SPIONs and their testing towards MHT.

## Conclusion

The heating potentiality of aqueous stable biocompatible Fe_3_O_4_ NPs coated with various surfactants such as GA, CA, PEG, PVP, EDA and CTAB with well-defined particle size, shape, magnetic anisotropy and susceptibility was successfully investigated. XPS asserted the presence of functional groups on the iron oxide core. MHT studies found that heating efficacy varied with different surface coatings due to effective anisotropy, relaxation time and magnetic susceptibility. Maximum magnetic susceptibility of 0.075 emu/gOe exhibited at HT temperature under magnetic field of 450 Oe leads to the highest SAR of 130 W/g in GA coated Fe_3_O_4_ NPs. In addition, shorter relaxation time and enhanced anisotropy also cause for maximum heat dissipation within short period. Moreover, this study pinpointed the fact that SAR is concentration dependent as exhibited by all functionalized NPs. The as synthesized NPs possessing enhanced heating efficacy proved its potential candidacy for future clinical MHT applications.

## Materials and methods

### Materials used

Ferric chloride (FeCl_3_·6H_2_O; ACS reagent, 97%), ferrous chloride (FeCl_2_·4H_2_O; puriss. p.a., ≥ 99.0%), ammonium hydroxide (NH_4_OH; extrapure AR, 25%), ethanol (99.9%), and surfactants such as GA (C_5_H_9_NO_4_; ≥ 99%), CA (C_6_H_8_O_7_; ACS reagent, ≥ 99.5%), PEG (H(OCH_2_CH_2_)_n_ OH-6000), PVP ((C_6_H_9_NO)_n_), EDA (NH_2_CH_2_CH_2_NH_2_; ≥ 99.5%) and CTAB (C_19_H_42_BrN; 99.0%) were procured from Sigma Private Ltd. As received analytical grades of chemicals were utilised and deionized (DI) water was used throughout the experiments.

### Synthesis of Fe_3_O_4_ NPs

Initially, 730 mg of FeCl_3_·6H_2_O and 270 mg of FeCl_2_·4H_2_O (Fe^3+^: Fe^2+^ in 2:1 molar ratio) were stirred until it gets dissolved completely in 50 ml of DI water. It was then heated to a temperature of 70 °C under constant magnetic stirring for a duration of 1 h. A black precipitate was formed on adding 30 ml of NH_4_OH to the above mixture. Further, 2 g of the respective surfactant (GA, CA, PEG, PVP, EDA and CTAB) previously dissolved in 5 ml DI water was added to the above iron oxide precipitate and raised the temperature to 90 °C and maintain for one more hour under constant stirring. Finally, the precipitated particles were thoroughly rinsed with DI water and ethanol for removing chloride ions and excess surfactants, and then collected by magnetic separation. The GA coated Fe_3_O_4_ is termed as GA-Fe_3_O_4_ and similarly for respective CA, PEG, PVP, EDA and CTAB coated Fe_3_O_4_ are termed respectively as CA-Fe_3_O_4_, PEG-Fe_3_O_4_, PVP-Fe_3_O_4_, EDA-Fe_3_O_4_ and CTAB-Fe_3_O_4_.

### Physical and chemical characterization

Crystalline structure was identified using X-ray diffraction (XRD) pattern using a diffractometer (D8 Advanced, Bruker) with a Cu-Kα radiation source (λ = 1.5406 Å, 40 kV). Debye–Scherrer method was used for the measurement of average crystallite size. Surface functionalization was characterized with the aid of Fourier transform infrared (FTIR) spectra recorded on a Shimadzu IR Prestige ranging from 400 to 4000 cm^−1^ on a Shimadzu IR Prestige. Thermogravimetric-differential thermal analysis (TGA) was performed by an SDT Q60 V20.9 thermal analyzer. For TGA, samples were heated from room temperature to 800 °C in a nitrogen atmosphere under a flow rate of 100 ml/min and a temperature ramp of 20 °C/min. Colloidal stability of MNPs in water was investigated by Horiba zeta potential analyzer and average of three values was calculated. High-resolution transmission electron microscopy (HRTEM) images and selected area diffraction (SAED) patterns were obtained using transmission electron microscope (TEM, Philips, Model: CM 200) operated under an accelerating voltage of 200 kV. Elemental composition and oxidation states of the surface species were analyzed by X-ray photoelectron spectroscopy (XPS) spectra obtained from Kratos AXIS ULTRA-DLD utilising Al Kα excitation source (14 kV). Magnetic measurements were conducted with the aid of a magnetic properties measurement system (MPMS-XL, Quantum Design).

### Evaluation of heating efficacy in terms of SAR

MHT measurements were carried out for determining the SAR using calorimetric method. Calorimetric HT was performed with the aid of a 4.2 kW Ambrell Easy heat 8310 system. Colloidal suspension of 1 mg/ml, 2 mg/ml and 3 mg/ml for all the prepared samples in water was used and the heating response was measured upon external magnetic field parameters of 450 Oe and 316 kHz. In order to prevent agglomeration between NPs, thorough sonication was performed for all samples prior to HT measurements. The temperature rise with respect to the exposure time for corresponding magnetic field parameters for all the samples was recorded. The heat dissipated in terms of SAR, expressed in W/g, was examined by the following formula:

$${\mathrm{SAR}}={\mathrm{C}}_{\mathrm{s}}.\frac{\Delta {\mathrm{T}}}{\Delta {\mathrm{t}}}. \frac{{\mathrm{M}}_{\mathrm{SOL}}}{{\mathrm{M}}_{\mathrm{MNPs}}}$$

where, ${\mathrm{C}}_{\mathrm{s}}$ is the specific heat capacity of solvent (C_water_ = 4.187 J/g℃); M_SOL_ and M_MNPs_ are masses of the solvent and MNPs used for measurement, and $\frac{\Delta \mathrm{T}}{\Delta \mathrm{t}}$ is the temperature–time dependent slope. An intrinsic parameter, intrinsic loss power (ILP), for power dissipation with respect to applied frequency (f) and amplitude (H) is also considered in case of calorimetric HT measurements i.e.,

$$\mathrm{ILP}=\frac{SAR}{f{H}^{2}}$$

This heat loss parameter denotes the efficacy of MNPs generating thermal energy from the applied electromagnetic energy.

## Supplementary information

Supplementary Information

## Data Availability

All data generated/analysed during the present work are included in this manuscript.
