# Supplementary material for: Assessing magnetic and inductive thermal properties of various surfactants functionalised Fe3O4 nanoparticles for hyperthermia
Source: Sci Rep. 2020 Sep 22;10:15045. doi: 10.1038/s41598-020-71703-6 (PMC7508873; doi:10.1038/s41598-020-71703-6)
Supplement: Supplementary file 1 — Supplementary Information [file 41598_2020_71703_MOESM1_ESM.docx]

**Supplementary Information**

**Assessing magnetic and inductive thermal properties of various surfactants functionalised Fe_3_O_4_ nanoparticles for hyperthermia**

Arunima Rajan S^1,2^, Madhulika Sharma^3^, Niroj Kumar Sahu^1*^


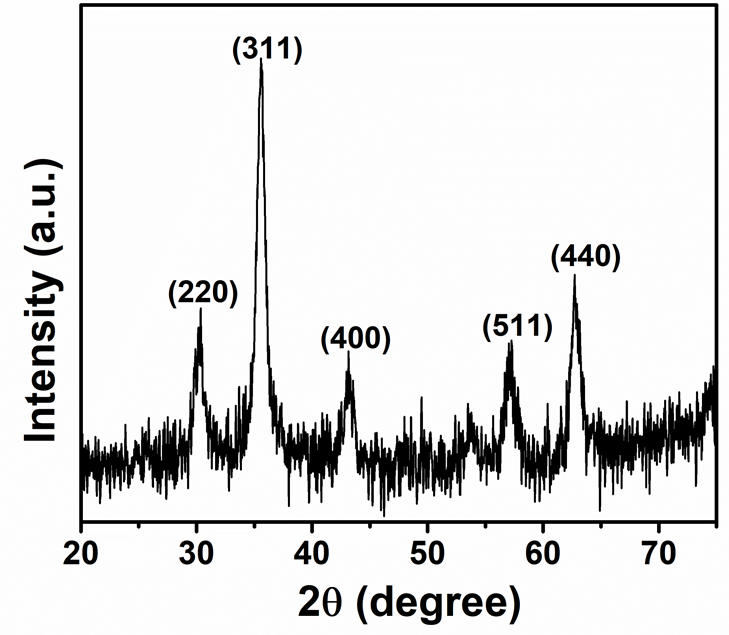


**Figure S1.** XRD pattern of uncoated Fe_3_O_4_ NPs.

**
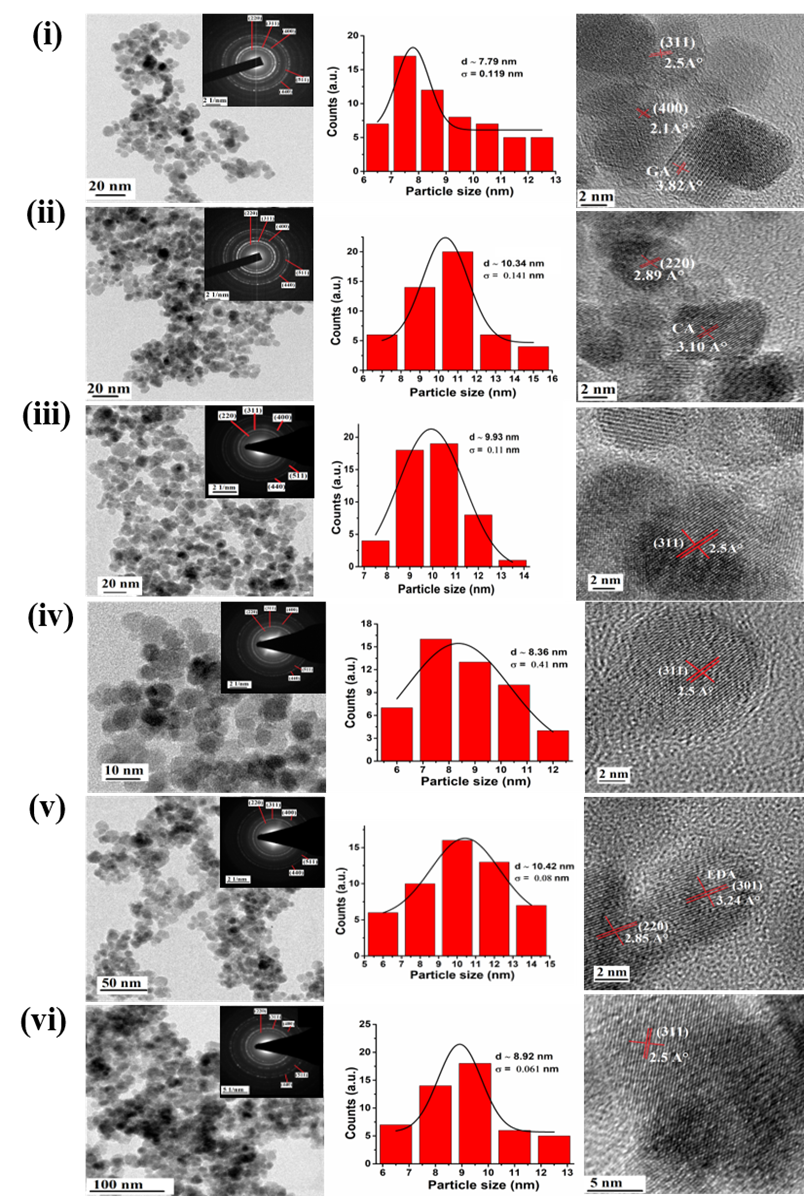
**

**Figure S2.** HRTEM image, particle size distribution (inset), lattice fringes and SAED of (i) GA-Fe_3_O_4_, (ii) CA-Fe_3_O_4_, (iii) PEG-Fe_3_O_4_, (iv) PVP-Fe_3_O_4_, (v) EDA-Fe_3_O_4_ and (vi) CTAB-Fe_3_O_4_ NPs.


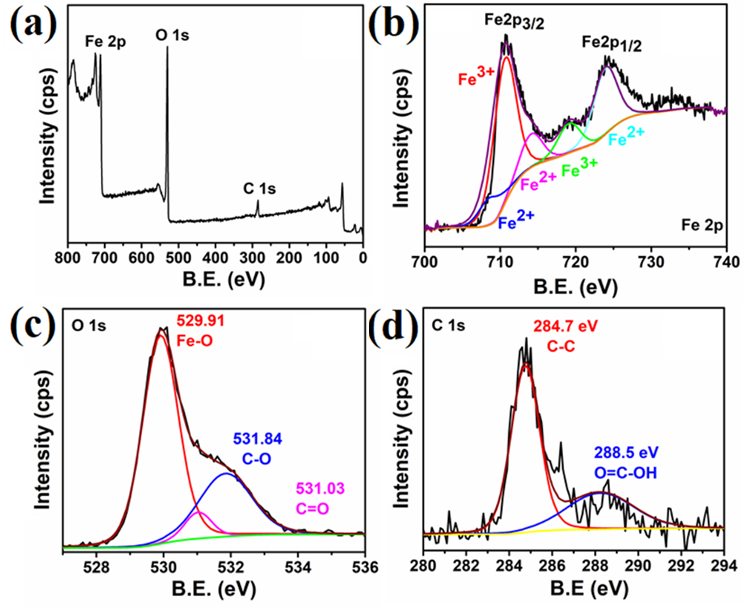


**Figure S3(i).** Deconvoluted XPS spectra of GA-Fe_3_O_4_ (a) fitted, (b) Fe 2p, (c) O 1s and (d) C 1s.


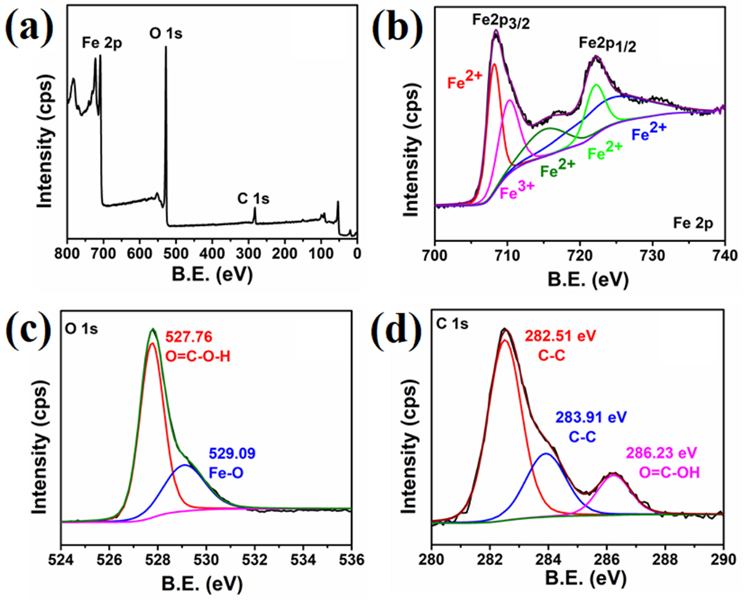


**Figure S3(ii).** Deconvoluted XPS spectra of CA-Fe_3_O_4_ (a) fitted, (b) Fe 2p, (c) O 1s and (d) C 1s.

In case of CA-Fe_3_O_4_ [Figure S3(ii)], five peaks were observed for Fe at 723.62, 722.05, 715.17, 710.16 and 708.13 eV. XPS profile of O1s electrons deconvoluted into two peaks located at 527.76 eV and 529.09 eV corresponds to the oxygen in an O-H or O-C components and oxygen species in Fe-O component of magnetite. From C 1s XPS spectrum, three peaks located at 282.51 and 283.91 eV corresponds to the C-C bond and 286.23 eV corresponds to the carbon atom attached to the different moieties (either oxygen or hydrogen).


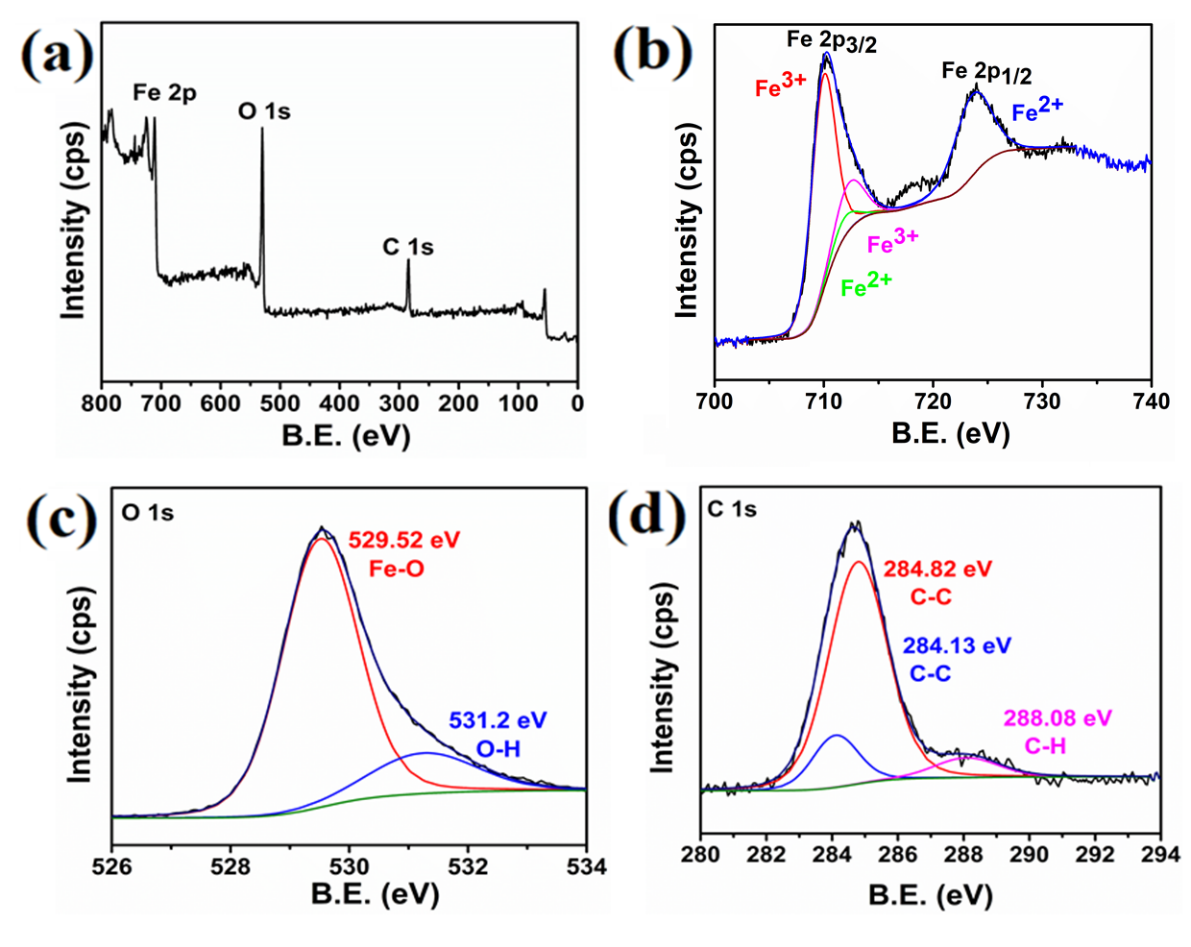


**Figure S3(iii).** Deconvoluted XPS spectra of PEG-Fe_3_O_4_ (a) fitted, (b) Fe 2p, (c) O 1s and (d) C 1s.

For PEG-Fe_3_O_4_ [Figure S3(iii)], prominent four peaks corresponding to Fe were observed at 723.9, 712.8, 711.7 and 710.2 eV. Major peaks at 710.2 and 723.9 eV in Fe 2p corresponds to the Fe^3+^ and Fe^2+^ octahedral cations respectively. XPS profile of O1s electrons exhibited two major peaks at 531.2 eV and 529.52 eV corresponds to the oxygen in an O-H component of PEG and oxygen species in Fe-O component of magnetite respectively. From C 1s XPS spectrum, two peaks located at 284.13 and 284.82 eV corresponds to the existence of adventitious carbon and C-C bond and a peak at 288.08 eV corresponds to the C-H bond.

**
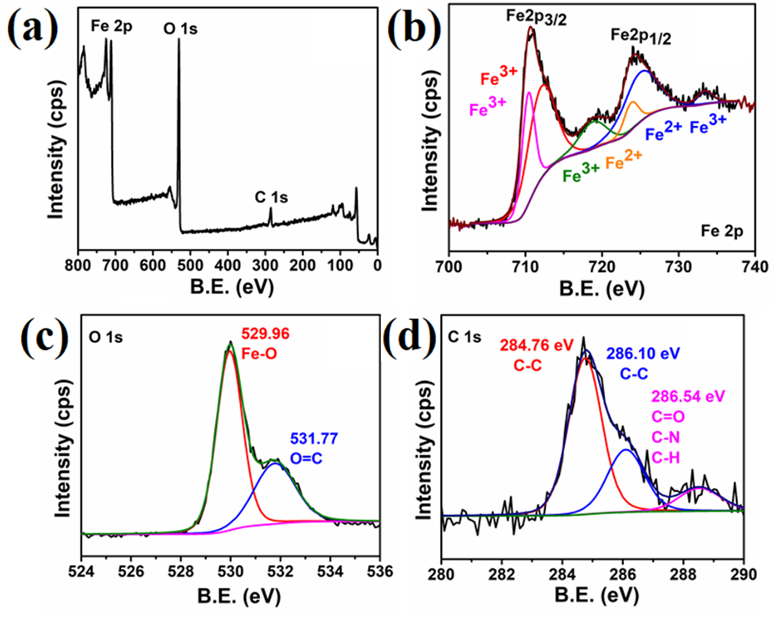
**

**Figure S3(iv).** Deconvoluted XPS spectra of PVP-Fe_3_O_4_ (a) fitted, (b) Fe 2p, (c) O 1s and (d) C 1s.

Similarly, for PVP-Fe_3_O_4_ [Figure S3(iv)], six peaks were observed for Fe at 724.95, 723.79, 718.77, 712.05,710.36 and 733.43 eV. An additional low intense peak at 733.43 eV attributed to the Fe 2p_3/2_ spectra seems similar to maghemite. XPS profile of O1s electrons deconvoluted in two peaks located at 529.96 eV and 531.77 eV corresponds to the oxygen species in Fe-O component of magnetite and carbonyl (C=O) oxygen in PVP. From C1s XPS spectrum, three peaks located at 284.76 and 286.10 indicated the C-C bonds and at 288.54 eV corresponds to the carbon atoms of pure PVP.


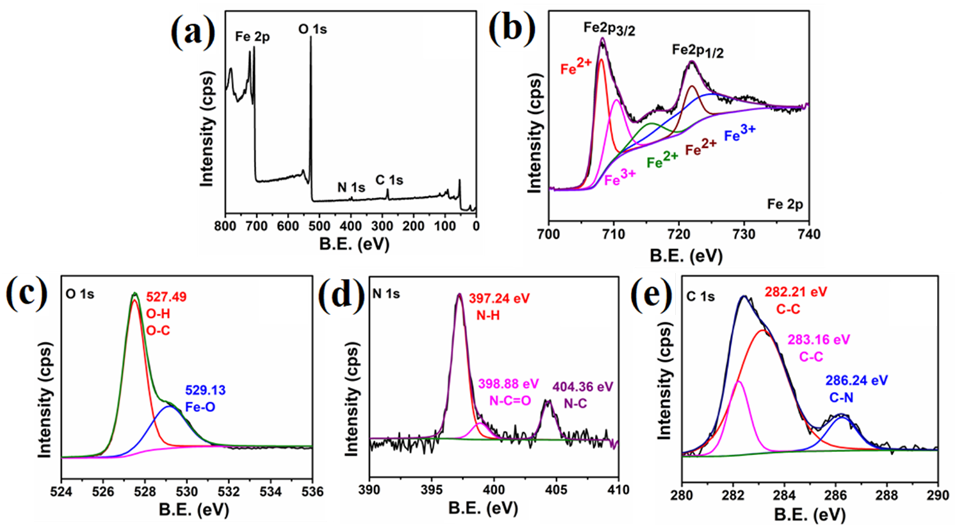


**Figure S3(v).** Deconvoluted XPS spectra of EDA-Fe_3_O_4_ (a) fitted, (b) Fe 2p, (c) O 1s (d) N 1s and (e) C 1s.

For EDA-Fe_3_O_4_ [Figure S3(v)], five peaks were observed for Fe at 722.61, 721.81, 715.3, 710.3 and 708 eV. XPS profile of O1s electrons deconvoluted in two peaks located at 527.49 eV and 529.13 eV corresponds to the oxygen species in an O-H, O-C components and oxygen species in Fe-O component of magnetite respectively. XPS profile of N1s core level spectra deconvoluted into three peaks located at 397.24 eV and 398.88 eV correspond to the N-H (nitrogen single bonded), amide carbonyl (N-C=O) which might be arising from NH_2_ binding states of EDA. Peak at 404.36 eV proven the existence of N-C. From C 1s XPS spectrum, two peaks located at 282.21 and 283.16 correspond to the C- C bond and third peak at 286.24 eV corresponds to C-N respectively. The C1s, N1s, O1sand Fe2p indicated the existence of EDA and Fe_3_O_4_.

**
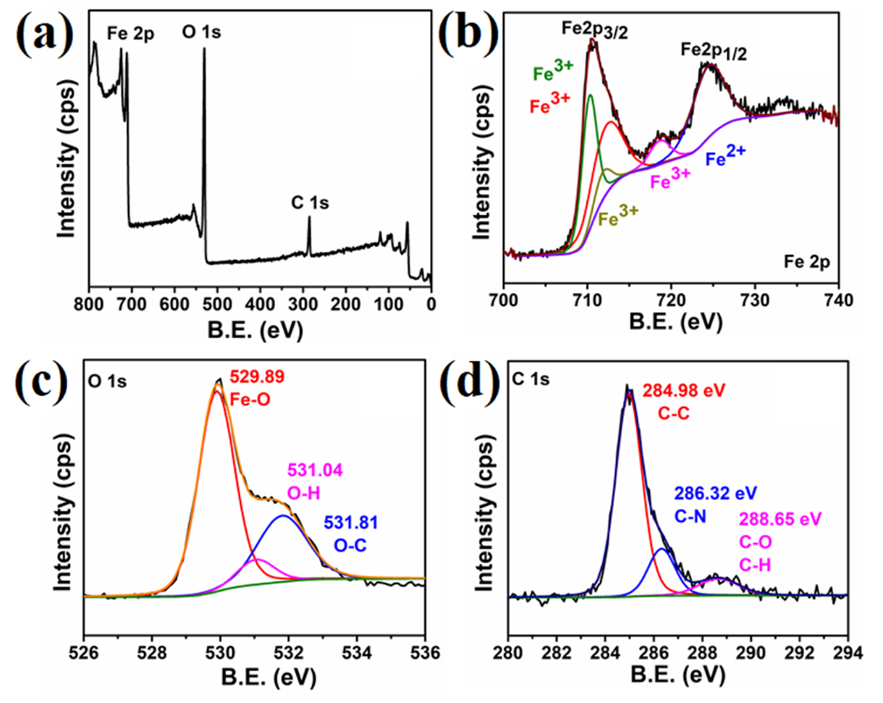
**

**Figure S3(vi).** Deconvoluted XPS spectra of CTAB-Fe_3_O_4_ (a) fitted, (b) Fe 2p, (c) O 1s and (d) C 1s.

For CTAB-Fe_3_O_4_ [Figure S3(vi)], five peaks were observed for Fe at 724.3, 718.6 (less prominent), 712.7, 711.8 (less prominent) and 710.2 eV. The satellite peak occurred at 718.6 eV corresponds to the Fe^3+^species of Fe_3_O_4_which does not overlap either the Fe 2p_3/2_ or Fe 2p_1/2_ peak indicating the oxidation of the iron complex. XPS profile of O1s electrons deconvoluted in three peaks located at 529.8, 531.04, and 531.8eV corresponds to the oxygen species in Fe-O, oxygen in an O-H component of ferric oxides as Fe-O-H and C-O respectively. From C 1s XPS spectrum, three peaks located at 284.98, 286.32 and 288.65 eV correspond to the aliphatic or alkyl carbon, C-N and O-C or C- H respectively.

Table S1. Binding energy values of the samples for XPS spectra.

| **Species present** | **Binding energies (eV) of** | | | | | |
| --- | --- | --- | --- | --- | --- | --- |
|  | GA-Fe_3_O_4_ | CA-Fe_3_O_4_ | PEG-Fe_3_O_4_ | PVP-Fe_3_O_4_ | EDA-Fe_3_O_4_ | CTAB-Fe_3_O_4_ |
| Fe 2p_1/2_ | 723.9 (Fe^2+^) | 723.62 (Fe^2+^)  722.05(Fe^3+^) | 723.9(Fe^2+^) | 724.95(Fe^2+^)  723.79(Fe^2+^) | 721.81(Fe^2+^)  722.61(Fe^3+^) | 724.34(Fe^2+^) |
| Fe 2p_3/2_ | 710.8 (Fe^3+^)  708.7 (Fe^2+^)  714.1 (Fe^2+^)  719.7 (Fe^3+^) | 710.16(Fe^3+^)  708.13(Fe^2+^)  715.17(Fe^2+^) | 710.2(Fe^3+^)  711.7(Fe^2+^)  712.8(Fe^3+^) | 710.36(Fe^3+^)  712.05(Fe^3+^)  718.77(Fe^3+^)  733.43(Fe^3+^) | 710.3(Fe^3+^)  708(Fe^2+^)  715.3(Fe^2+^) | 710.22(Fe^3+^)  711.9(Fe^3+^)  712.7(Fe^3+^)  718.6(Fe^3+^) |
| O 1s | 529.91  531.03  531.84 | 527.76  529.09 | 529.52  531.2 | 529.96  531.77 | 527.49  529.13 | 529.8  531.04  531.8 |
| C 1s | 284.26  288.5 | 282.51  283.91  286.23 | 284.13  284.82  288.08 | 284.76  286.10  288.54 | 282.21  283.16  286.24 | 284.98  286.32  288.65 |
| N 1s |  |  |  |  | 397.24  398.88  404.36 |  |


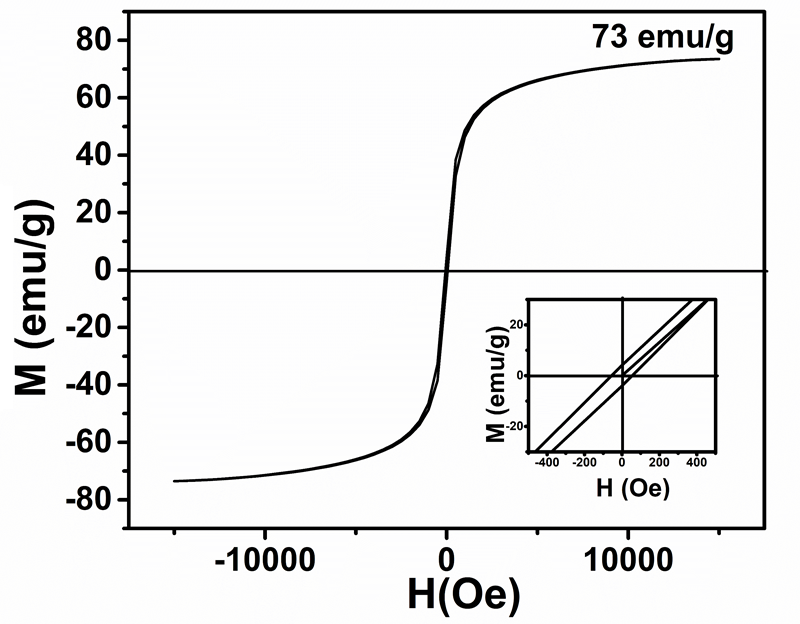


**Figure S4.** M-H curve of uncoated Fe_3_O_4_ NPs at 300 K.


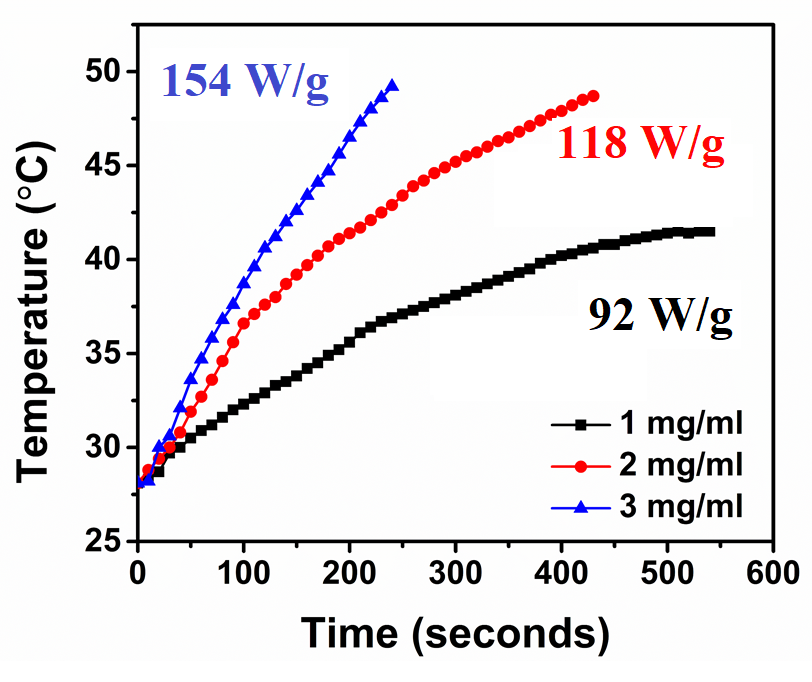


**Figure S5.** Temperature-time profiles for the colloidal dispersions of uncoated Fe_3_O_4_ for concentrations 1 mg/ml, 2 mg/ml and 3 mg/ml under magnetic field parameters of 35.28 kA/m and 316 kHz.


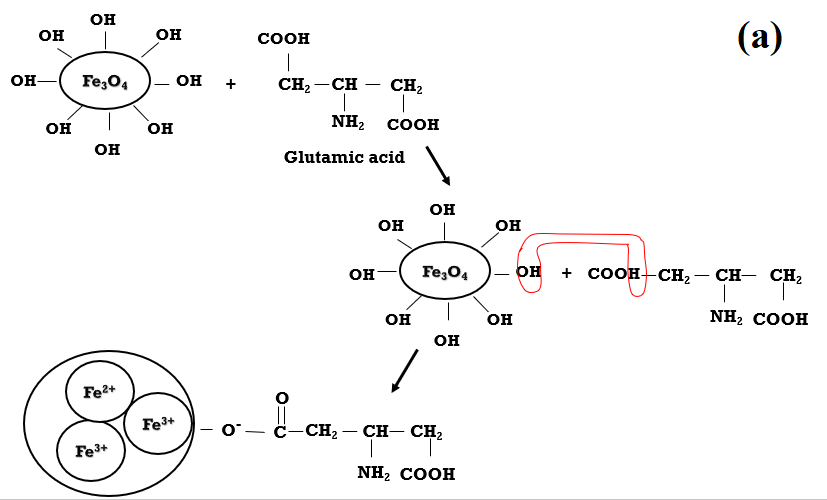


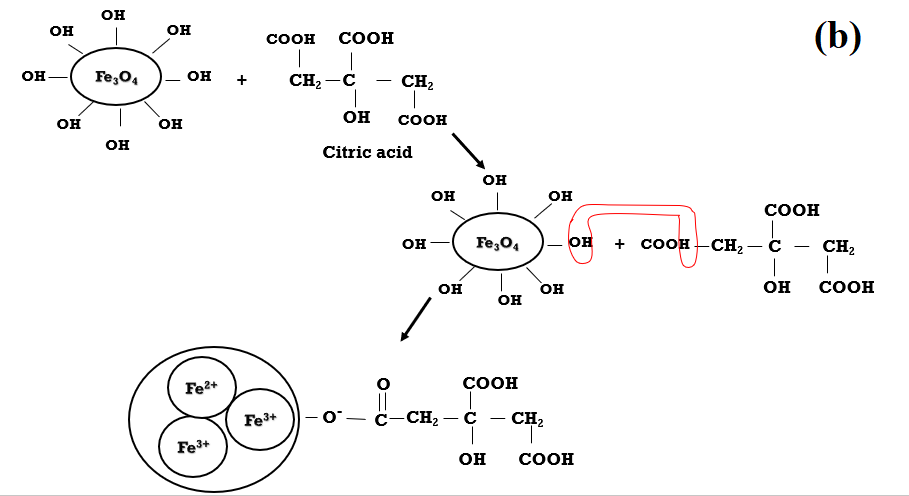


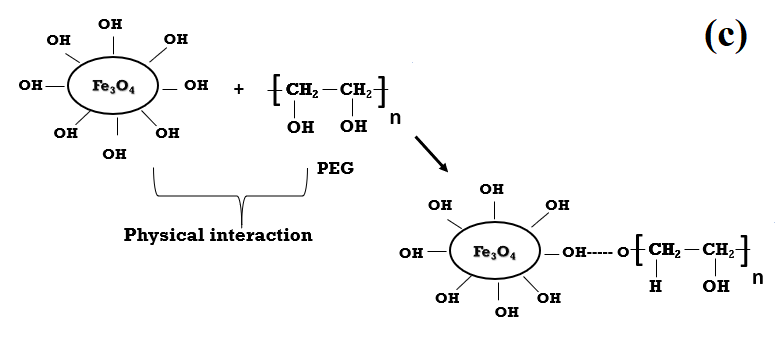


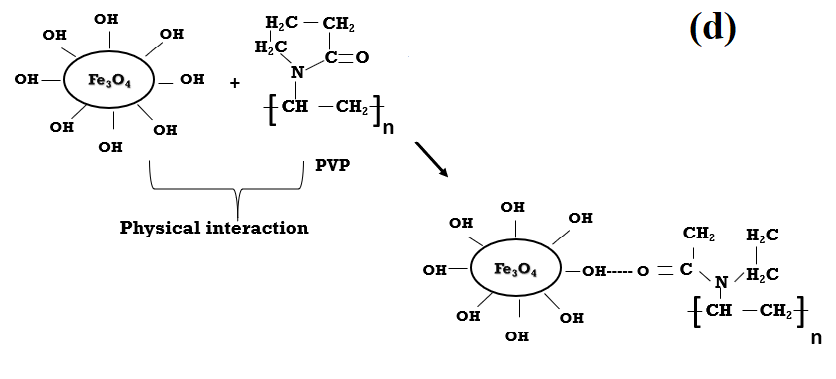


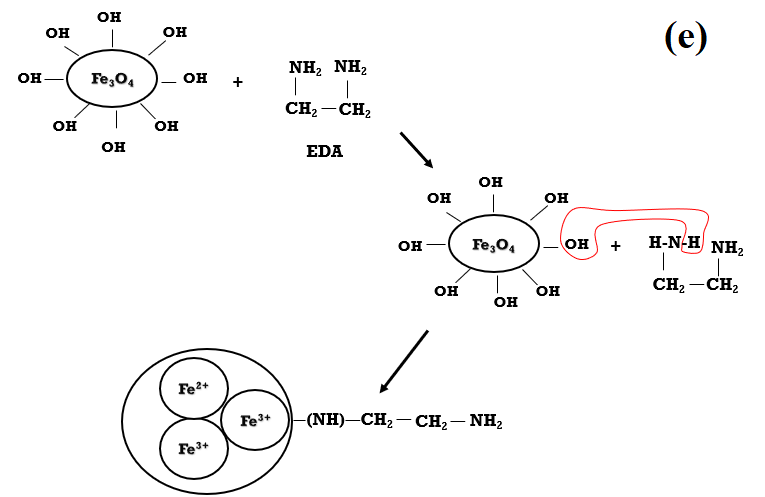


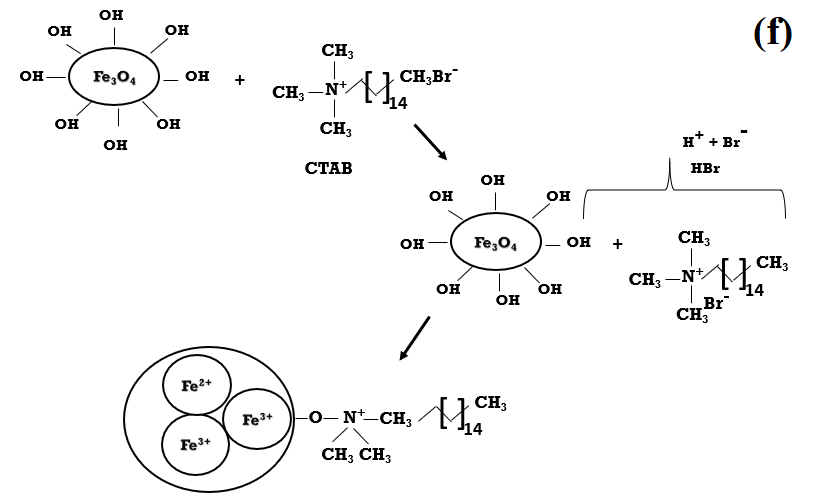


**Figure S6.** Schematic of the surface binding interaction of different surfactant molecules on Fe_3_O_4_NPs (a) GA- Fe_3_O_4_, (b) CA- Fe_3_O_4_, (c) PEG- Fe_3_O_4_, (d) PVP- Fe_3_O_4_, (e) EDA- Fe_3_O_4_ and (f) CTAB- Fe_3_O_4_ NPs.

**Table S2.** Details of the magnetic data calculated from the M-T curves of the as-synthesized MNPs.

| Sample | Maximum M_s_ (emu/g) | Blocking temp  (T_B_) (K) | Anisotropy energy constant (K)  (x 10 ^5^erg/cm^3^) |
| --- | --- | --- | --- |
| GA-Fe_3_O_4_ | 35 | 164 | 2.05 |
| CA-Fe_3_O_4_ | 37 | 124 | 1.76 |
| PEG-Fe_3_O_4_ | 34 | 138 | 1.68 |
| PVP-Fe_3_O_4_ | 33 | 121 | 1.72 |
| EDA-Fe_3_O_4_ | 33 | 128 | 1.21 |
| CTAB-Fe_3_O_4_ | 34 | 146 | 1.33 |
